# Bridging agronomic science and context specific farm-level advisory through generative AI for rice systems in India

**DOI:** 10.3389/frai.2026.1808028

**Published:** 2026-07-29

**Authors:** Shalini Gakhar, Jawoo Koo, Girija Prasad Patnaik, Raj Kumar Singh

**Affiliations:** 1 International Rice Research Institute, New Delhi, India; 2 International Food Policy Research Institute, Washington, DC, United States

**Keywords:** agro-advisory, crop simulation, digital agriculture, generative AI, large language models

## Abstract

Agriculture is increasingly characterized by a data paradox, while the sector generates massive volumes of genomic, climatic, remote sensing, and field data. Translating this information into actionable, farm-level insights remains a critical bottleneck. Traditional advisory mechanisms cannot operate at the spatial scales or provide the context-specificity needed for climate adaptation and food security. The work presents GenAI as a transformative interface that makes advanced agricultural science, including crop simulation models and remote sensing diagnostics, accessible to rice farmers in India through natural language, provided that challenges related to data sovereignty, infrastructure gaps, and the need for human oversight in advisory systems are effectively addressed. We trace the shift from static, rule-based expert systems to dynamic foundation models capable of complex reasoning and multimodal analysis. The paper critically reviewed the rise of crop-specific LLM agro-advisory model architectures, such as SeedLLM-Rice and IPM-AgriGPT, which outperform general-purpose models by utilizing specialized scientific corpora to reduce hallucinations. Main attention is on the hybrid integration framework of LLMs with Knowledge Graphs (KGs) for factual grounding, and connecting with process-based simulators (e.g., DSSAT, APSIM) to make biophysical modelling more accessible through natural language. Furthermore, the manuscript examines the fusion of satellite, drone, and smartphone imagery with vision-language models, enabling real-time, context-aware diagnostics for smallholder farmers. The review concludes that while GenAI has the potential to support more equitable forms of precision agriculture, it can only do so under specific conditions. Its success depends on resolving infrastructure gaps, ensuring data sovereignty, and maintaining a “human-in-the-loop” architecture to guarantee scientific rigour and social inclusion.

## Graphical abstract

**Figure:**
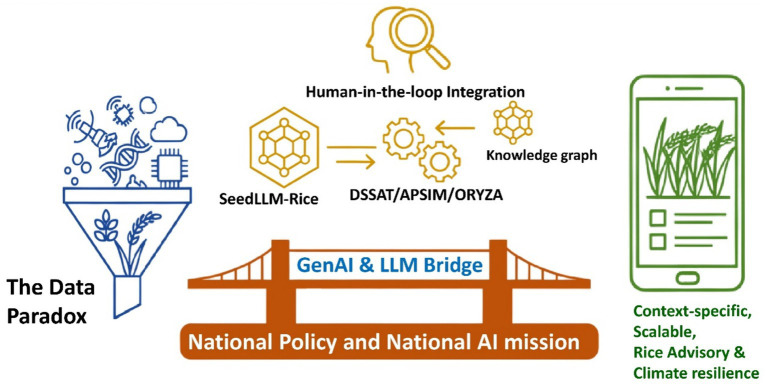
GenAI-enabled bridge connecting fragmented agricultural data with context-specific rice advisory through foundation models, knowledge graphs, and human-in-the-loop integration.

## 1 Introduction

### 1.1 Background

Agriculture is operating under the combined pressures of ensuring food security, adapting to climate variability, and reducing environmental impacts, which will shape food systems in the coming decades (Sapkota et al., 2025). By 2050, the world’s agricultural systems will need to grow food for an estimated 10 billion people apart from adapting to unpredictable changes in climate patterns and achieving sustainable intensification that preserves natural resources and the environment (Wise, 2013). This convergence of demographic pressure, environmental uncertainty, and sustainability imperatives demands transformative innovation in how agricultural knowledge is generated, disseminated, and applied across diverse farming contexts. Traditional agricultural advisory systems face fundamental limitations despite their historical contributions (Manimozhi and Krishnamoorthy, 2025). Extension services operate at a limited scale with delayed responses to emerging threats due to low advisor-to-farmer ratios (Bonilla et al., 2024). Generic recommendations fail to address diverse farm conditions, particularly affecting underserved smallholder farmers in developing regions (Tumwebaze et al., 2025; Prajapati et al., 2025). The digital revolution has transformed agriculture’s data landscape through IoT sensors, satellite imagery, and genomic sequencing (Birner et al., 2021; Marudamuthu et al., 2023). However, translating these massive, varied datasets into practical, actionable insights for farmers remains extraordinarily difficult.

Generative AI (GenAI) fundamentally differs from traditional machine learning approaches in agriculture (Sai et al., 2025). While conventional systems focus on prediction and classification, including forecasting yields, detecting diseases, and optimizing irrigation (Paudel et al., 2021; Bharadiya et al., 2023), GenAI employs large language models (LLMs), diffusion models, and generative adversarial networks to generate content, facilitate reasoning, and enable natural conversations (Shaikh et al., 2025; Yang et al., 2025a). Unlike standard deep learning models that merely estimate disease likelihood from images, GenAI systems engage farmers conversationally, explain reasoning, integrate multi-source knowledge, provide personalized advice, and generate synthetic training data for data-limited conditions (Sai et al., 2025; Chirra et al., 2025).

India’s rice production faces critical sustainability challenges that threaten the nation’s food security (Gathorne-Hardy et al., 2016). Kumar et al. (2021) documented that long-term intensive rice cultivation in northern Indian plains has led to severe deterioration of natural resources, declining factor productivity, multiple nutrient deficiencies, depleting groundwater, and labor scarcity. The rice-wheat cropping system in the Indo-Gangetic Plains shows particularly alarming trends, with groundwater tables declining approximately one meter per year while crop productivity stagnates (Khedwal et al., 2023). Rice demand in India is expected to rise sharply by mid-century, while resource degradation and environmental stresses threaten sustainable production. GenAI presents transformative solutions to address these multifaceted challenges. The AgriLLM initiative, developed by Consultative Group on International Agricultural Research (CGIAR) in partnership with AI71, provides region-aware, role-specific responses supporting farmers, advisors, and decision-makers with multilingual capabilities (CGIAR, 2025). GenAI is not here to replace what farmers already know, but to be a powerful helper that makes expert farming advice accessible to everyone. It breaks down technical barriers, allowing small farmers who may not read well or use technologies to ask questions in their own language and receive informed answers (Kumar et al., 2025; Sai et al., 2025; Chirra et al., 2025). It does not replace local agricultural advisors, but it helps them reach more farmers more quickly with up-to-date information and tools.

### 1.2 Scope and objectives

This review addresses the central research question: What is the current landscape of generative AI applications for rice systems in India, and how do these applications bridge agronomic science with farm-level advisory delivery? This scoping review establishes clear boundaries, providing focused yet comprehensive coverage of generative AI applications in agriculture and food systems. While the agricultural challenges faced are universal, this analysis examines global trends with a particular focus on rice production systems. Rice serves as the staple food for more than half of humanity, predominantly in Asia, Africa, and Latin America, where smallholder farming remains the dominant production model (Asma et al., 2023; Mohapatra et al., 2025). The recent development of SeedLLM, a specialised large language model for rice cultivation, exemplifies how crop-specific AI systems are emerging to address the unique agronomic complexities of individual commodities (Lu et al., 2024; Yang et al., 2025a). This focus allows us to explore both the technical innovations and practical implementation challenges that characterize the frontier of agricultural AI development.

The geographic scope of this review is centred on Indian agricultural contexts but draws on international evidence where Indian-specific literature is sparse, including from related institutional bodies such as the International Rice Research Institute (IRRI), CGIAR, and the Food and Agriculture Organization (FAO) CABI (2024). Of the 183 sources included in the scoping synthesis, 25.7% directly address Indian agricultural contexts, while the remainder contribute methodological, architectural, or international policy perspectives that inform a synthesis with implications for Indian smallholder rice systems. This India-centred framing with transferable international evidence is consistent with the realities of an emerging research field in which crop-specific generative AI work for any single national context remains, by necessity, a small fraction of the global literature.

Temporally, this work concentrates on the transformer era in AI, beginning with the architectural breakthroughs that enable modern LLMs and extending through the present moment. Since around 2017, model capabilities have proliferated, foundation models have emerged, and methods for domain adaptation have advanced significantly. By focusing on this transformative period, the analysis demonstrates how advances in general-purpose AI were adapted and refined for agricultural applications. Earlier rule-based expert systems and traditional machine-learning methods are mentioned only to provide historical context and to highlight the major shift introduced by generative artificial intelligence.

This manuscript outlines five objectives illustrating agricultural AI development and future directions. It traces evolution from early rule-based systems through machine learning, deep learning, to modern generative AI, emphasizing how innovations have transformed agricultural knowledge while highlighting risks of losing valuable earlier approaches. The review examines crop-specific LLMs like SeedLLM for rice versus broad foundation models, discussing domain adaptation, data requirements, and specialization trade-offs. It presents integration frameworks combining AI tools, knowledge graphs, sensing systems, and crop simulation models, and acknowledging development complexities. The global policy environment is evaluated, with particular emphasis on India’s agricultural AI, offering lessons on regulation, investment, digital access, and smallholder farmer benefits. Finally, it identifies transformative opportunities like personalized advisory systems and accelerated crop improvement, while addressing challenges including data gaps, infrastructure limitations, technical issues, and adoption barriers. Together, these objectives map the current landscape of generative AI in agricultural advisory, expose integration gaps across sensing, knowledge, model, interface, and governance layers, and identify the institutional and policy questions that will shape responsible deployment in Indian rice systems.

## 2 Materials and methods

**Figure 1:**
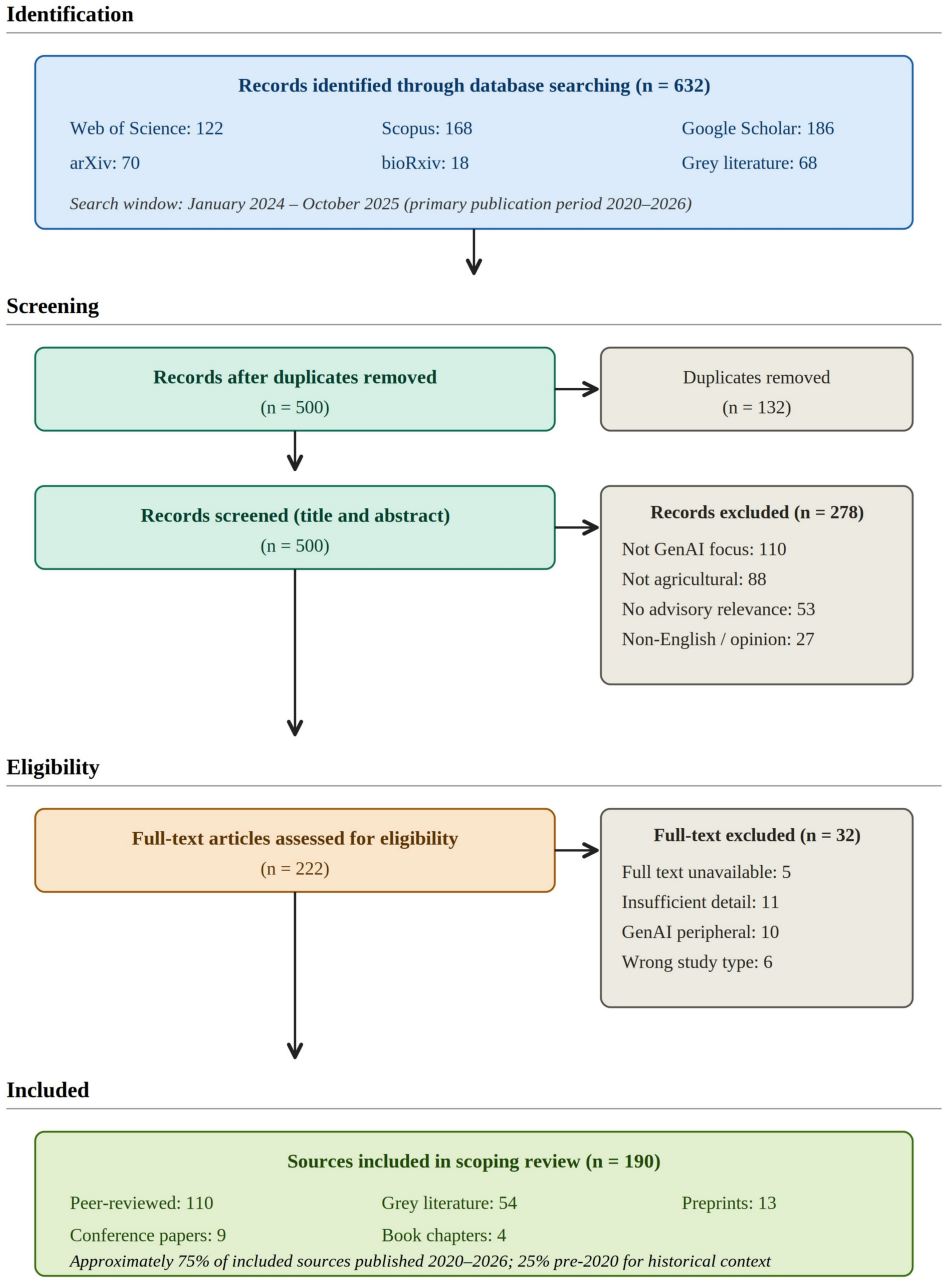
PRISMA-ScR flow diagram showing the identification, screening, eligibility, and inclusion of sources in the scoping review.

In this scoping study, a comprehensive search approach has been employed to encompass both formal research studies and practical examples of generative AI in agriculture. Because the field is advancing at an exponential rate, important innovations often appear first on preprint servers, blogs, or open-source platforms before they are published in peer-reviewed journals. This indicates that our approach extends beyond conventional academic databases to capture the full range of relevant evidence in this fast-moving field. We conducted structured searches across multiple complementary databases to ensure comprehensive coverage of the literature. Web of Science and Scopus provided access to established peer-reviewed literature, capturing research that has undergone formal quality assessment. Google Scholar has extended its reach to include conference proceedings, technical reports, and publications from regional journals that may not be indexed in primary services. To maintain reproducibility, Google Scholar results were limited to the top 180 relevance-ranked records, consistent with established scoping review practice (Haddaway et al., 2015). Recognizing that cutting-edge AI research often appears first as preprints, we systematically searched arXiv’s computer science and quantitative biology sections, as well as bioRxiv, for agricultural applications with computational components. This multi-database approach enabled coverage across formally vetted academic literature, emerging preprint research, and grey literature (Figure 1).

The keyword strategy struck a balance between specificity and comprehensiveness to identify relevant literature. Core search terms included “large language model agriculture,” “generative AI crop,” “agricultural knowledge graph,” “multimodal agricultural AI,” and “crop simulation LLM.” The related terms-, such as “foundation model farming,” “transformer agriculture,” “conversational AI advisory,” “diffusion model crop breeding,” and crop-specific queries like “rice language model” and “wheat phenotype generation,” were supplemented. Named-system queries were also included to capture specific crop-adapted models reported in the field, such as “AgriLLM,” “AgriGPT,” “SeedLLM,” “AgriLLaVA,” “AgroLLM.” Boolean operators enabled the combination of domain terms (such as agriculture, farming, crop, and agronomy) with AI technology terms (LLM, generative AI, GPT, transformer, and diffusion model) to capture interdisciplinary work. The study primarily focused on literature from 2020 to 2026, a period marked by rapid growth in generative AI capabilities and agricultural applications. This timeframe encompasses the transition from early transformer models to sophisticated foundation models, as well as their domain adaptation for agricultural applications. Selected literature from 1980 to 2019 provides essential background on the evolution from rule-based expert systems to conventional machine learning approaches and establishes the historical context necessary to understand the paradigm shift that generative AI represents. Grey literature formed a crucial component of the evidence base, reflecting the reality that much innovation in applied AI occurs outside traditional academic publishing. Systematically reviewed reports from bodies such as the Ministry of Agriculture and Farmers Welfare, the Department of Science and Technology, Government of India, and the Indian Space Research Institute, which capture policy perspectives and national strategic initiatives, are also part of the detailed scoping.

Sources were eligible for inclusion if they addressed generative AI, large language models, foundation models, diffusion models, or knowledge graph–integrated systems with relevance to agriculture, with priority given to rice, related cereals, and Indian agricultural contexts. Peer-reviewed journal articles, conference proceedings, substantive preprints, book chapters, and institutional grey literature were all included. Studies addressing agricultural AI without generative or foundation-model components, opinion pieces lacking technical substance, non-English publications, and duplicate records were excluded.

Records identified across all sources were exported and de-duplicated, after which a two-stage screening process was applied: title and abstract review against the eligibility criteria, followed by full-text assessment of records passing the first stage. Discrepancies were resolved through discussion among the authors. A total of 632 records were identified, of which 500 remained after duplicate removal. Title and abstract screening excluded 278 records, and a further 32 were excluded at full-text review, yielding 190 sources included in the scoping synthesis. The complete screening process is summarized in Figure 1.

During synthesis, included sources were organized according to a consistent set of characteristics to enable thematic comparison across the evidence base. These characteristics included publication type, the primary AI architecture or approach, the target crop and agricultural task, geographic focus, the stage of evaluation reported (distinguishing benchmark performance, experimental evaluation, and real-world deployment), and the principal limitations noted. Consistent with PRISMA-ScR guidance (Tricco et al., 2018), formal critical appraisal of individual sources was not undertaken, as the review aims to map the breadth of evidence and identify thematic patterns rather than to evaluate study-level methodological quality. Given the heterogeneity of the included sources, a narrative synthesis approach was adopted, supplemented by structured comparative tables and an integrative conceptual framework.

## 3 An integrative framework for generative AI in rice advisory

Generative AI in agriculture is usually described as a series of separate advances: a crop-specific language model, a knowledge graph, a sensing pipeline, a chatbot, a policy paper. Each is useful alone, but the literature says little about how they connect into a working advisory system. A farmer benefits only when data from the field is structured, reasoned over, and returned as usable guidance. To organise the synthesis that follows, we propose a framework that treats the major components of the field as interdependent layers of a single architecture.

**Figure 2:**
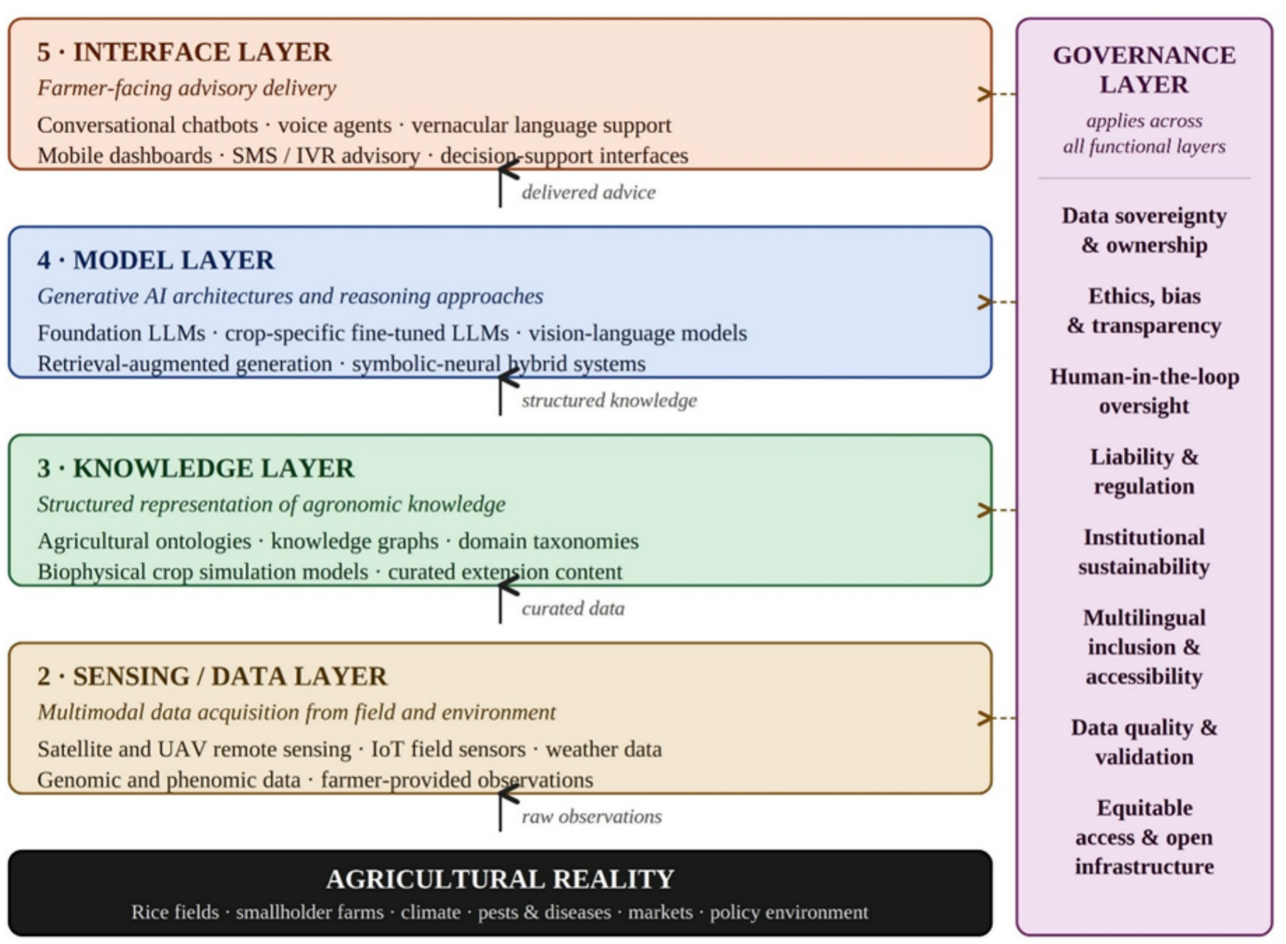
Integrative five-layer conceptual framework for generative AI in rice advisory systems.

As shown in Figure 2, the framework has four functional layers grounded in agricultural reality, with a governance layer applying across all of them. The sensing layer acquires multimodal data from the field, including remote sensing, IoT sensors, weather, genomics, and farmer observations. The knowledge layer structures this data through ontologies, knowledge graphs, crop simulation models, and curated extension content, encoding the agronomic logic that separates informed advice from generic text. The model layer reasons over this knowledge using foundation LLMs, crop-specific models, vision-language models, retrieval augmented generation, and hybrid systems. The interface layer delivers the result to farmers through chatbots, voice agents, vernacular support, and mobile channels, and is decisive for smallholder impact since even the strongest model is useless if its output cannot be accessed and trusted. The governance layer runs alongside all four, mediating data sovereignty, ethics, oversight, liability, institutional sustainability, inclusion, and validation at every stage.

**Figure 3:**
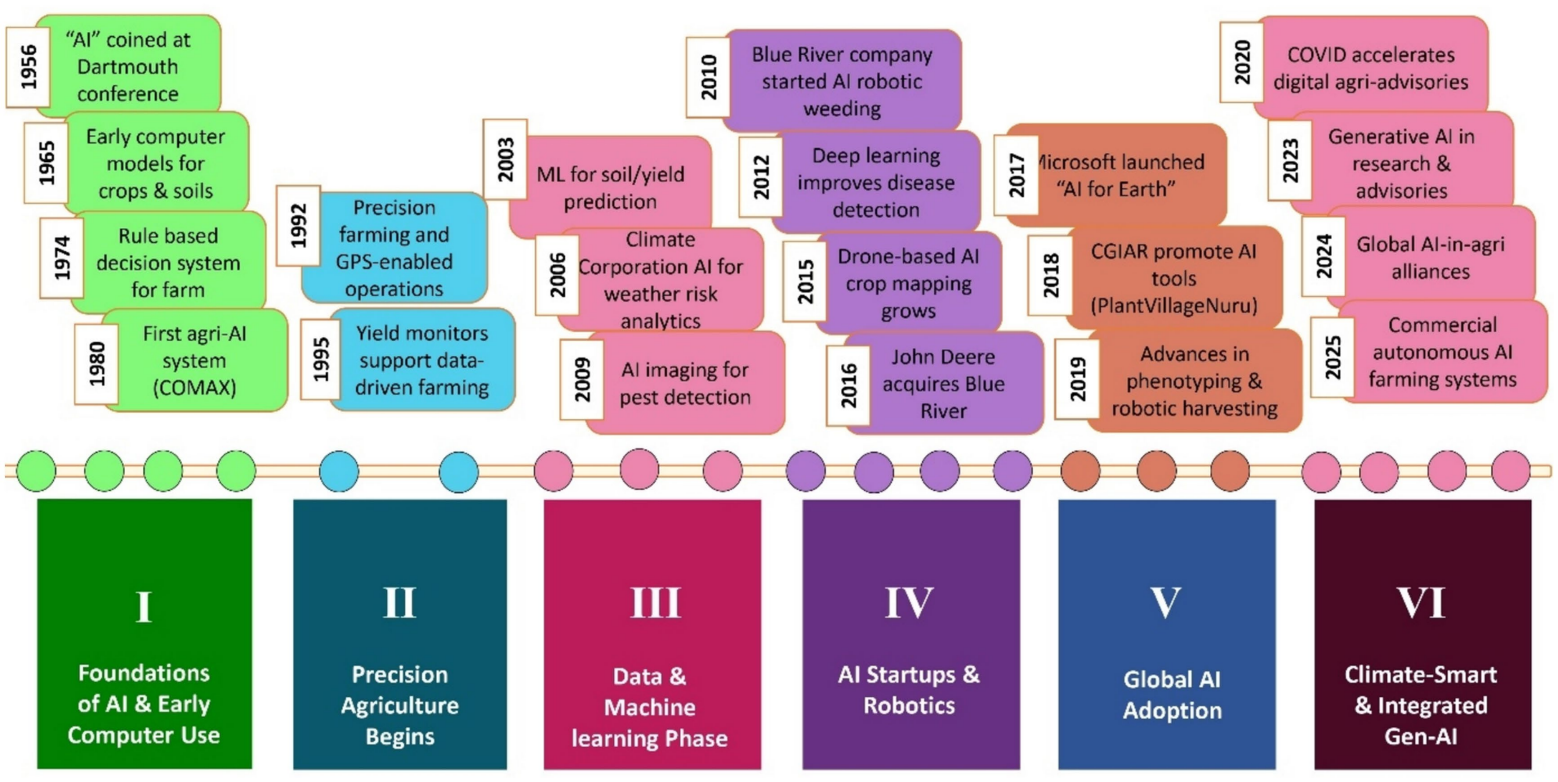
Timeline of GenAI’s evolution in agriculture, along with subsequent developments.

The rest of this review is organized around these layers, treating each not as an isolated topic but as part of the architecture in Figure 3. This lens also surfaces the review’s central finding: research is unevenly distributed across the layers, and the most consequential gaps lie not within any single layer but in the bridges between them, particularly between sensing and knowledge, between models and structured knowledge, and between technical capability and validated impact at the farmer interface.

## 4 The evolution of agricultural AI

### 4.1 From rule-based decision support systems (DSS) to learning systems

Over the past 70 years, AI in agriculture has transformed from basic rule-based systems to data driven learning methods and now to autonomous tools powered by foundation models.

AI was coined at the 1956 Dartmouth Conference (McCarthy et al., 2006). The 1970s brought rule-based expert systems, with 1980s tools like COMAX for cotton and POMME for apple orchards translating expertise into decision rules (Lemmon, 1986; Roach et al., 1985). Early 1990s precision agriculture introduced GPS equipment and yield monitors, but knowledge capture remained expensive and systems struggled with unexpected weather or pests (Feigenbaum, 1984; McCown et al., 1996). Between 2000–2015, machine learning shifted from rigid rules to data patterns, with Climate Corporation’s 2006 weather tools and 2003 soil projections (Drummond et al., 2003). Random Forests and Support Vector Machines improved yield predictions (Dang et al., 2021), while deep learning tackled disease detection by 2012 and IBM Watson analyzed agricultural data by 2013. Post-2015, deep learning accelerated with drones mapping crops (2015), John Deere’s smart machinery acquisition (2017), and CNNs excelling at disease identification using PlantVillage data (Pandey et al., 2024). Cloud platforms like Microsoft’s AI for Earth (2017) and smallholder apps emerged (Mrisho et al., 2020). By 2020, foundation models and transformers revolutionized agriculture (Paul and Chen, 2022), with LLMs offering multilingual guidance and IoT enabling real-time monitoring. As climate-smart solutions and autonomous farms reach 2026 markets, challenges including bias, opacity, and digital divides demand collaboration among researchers, farmers, and policymakers (Manvi et al., 2023).

### 4.2 Shifts in data availability: sensors, remote sensing, genomics, and digitisation

Agriculture has undergone significant changes over the past decade, driven by the rapid growth of data. This change has been driven by sensors, remote sensing tools, plant genomics, and digital platforms (Shakoor et al., 2019). These data sources now support modern decision systems, improving accuracy and efficiency in farming. Sensors and IoT devices collect real-time information on soil moisture, temperature, nutrients, and microclimate (Sehrawat and Gill, 2019). Farmers use these measurements to plan irrigation and fertiliser use precisely. Intelligent sensor networks and wireless devices allow continuous monitoring to detect soil or crop stress early (Tyagi et al., 2020). With edge computing, data can be analysed directly in the field to predict nutrient deficiencies or disease outbreaks (Bondi-Kelly et al., 2023; Nikitha et al., 2025). Remote sensing using satellites and drones provides detailed maps of crop health, water status, and biomass. Combining drone and satellite data improves nitrogen estimation, yield prediction, and stress detection (Pan et al., 2025; Chatraei Azizabadi et al., 2025). This helps apply inputs only where required, reducing waste and improving sustainability. Advances in plant genomics have also sped up breeding progress. Sequencing technologies and genome-wide markers facilitate the identification of functional genes for yield, stress tolerance, and disease resistance (Yunus et al., 2025; Xue and Cui, 2025). The digitisation of field records, weather data, and soil profiles has enhanced knowledge sharing (Seethapathy et al., 2025). Growing data availability from sensors, remote sensing, genomics, and digitisation enables precise, timely, and scalable farm decisions. These rich, accessible datasets accelerate crop improvement, support knowledge sharing, and provide the essential foundation for advanced analytics and agriculture-specific AI models that generate reliable, context-aware insights for farmers and policymakers.

### 4.3 Rise of domain-specific language models (Agri LLMs)

AI has advanced rapidly over the past decade, significantly improving how computers read, understand, and generate human language. General LLMs, such as GPT, LLaMA, and PaLM, perform well across many fields; however, their performance declines in highly specialised, knowledge-intensive areas, such as agriculture (Shaikh et al., 2025). This challenge has encouraged the development of domain-specific models, particularly Agri LLMs (Sapkota et al., 2025), that integrate deep agricultural knowledge with modern AI methods. The rise of Agri LLMs signals a significant shift in digital agriculture, creating new opportunities to enhance decision-making, strengthen advisory systems (Tzachor et al., 2023), and expand access to reliable scientific knowledge across the entire agri-food sector.

The integration of diverse climate and crop data becomes easier when information from real-time weather forecasts, long-term climate projections, soil health indicators, pest surveillance, and crop management records is combined into a single system (Patil et al., 2023; Yu et al., 2025). This unified data flow supports rapid, climate-smart advisories tailored to specific crops, regions, and seasons.

Crop models, such as DSSAT (Decision Support System for Agrotechnology Transfer), APSIM (Agricultural Production Systems Simulator), and ORYZA, become easier to use when key variables are chosen automatically, and parameters are adjusted for a changing climate, allowing even non-experts to run these simulations (Gavasso-Rita et al., 2024). Early warning and climate-risk assessment improve as climate signals are linked with historical crop performance to predict threats and issue timely alerts. Yield forecasts become more accurate and interpretable by fusing structured and unstructured data using machine-learning methods (Van Klompenburg et al., 2020; Rashid et al., 2021). Decision support also becomes clearer, with simple explanations of yield drivers and adaptive management tips in the local language. Multilingual chatbots, voice tools, and SMS help scale advisory services to millions of farmers, while continuous feedback strengthens both model performance and climate-smart recommendations over time (Ittarat et al., 2023; Tzachor et al., 2023).

Domain-specific LLMs are a crucial step in agricultural AI, as they are designed to better handle the unique and complex needs of crop science and agronomy than general models (Song et al., 2025; Ling et al., 2025). These specialised models address a key weakness of general LLMs: models such as GPT-4 and Claude possess vast knowledge, but they lack the deep, precise, and technical understanding of agricultural concepts needed for expert reasoning in crop biology, pest control, and farming systems (Yang et al., 2024). SeedLLM-Rice is a pioneering example, representing a 7-billion-parameter model trained from scratch on 1.4 million rice-related research publications, encompassing nearly 98% of the global literature on rice biology (Yang et al., 2025a). Developed by researchers at China Agricultural University and the Shanghai Artificial Intelligence Laboratory, The model’s integration with the Rice Biological Knowledge Graph, which consolidates genome annotations and synthesises transcriptomic and proteomic information from over 1,800 studies, enables it to address complex research questions that require the fusion of textual and multimodal data.

Agricultural AI development features specialized models like AgriGPT (Qwen3-8B with Agri-342 K dataset), AgriLLM (handling 4 M Indian farmer queries), and AgroLLM (93% accuracy using RAG with ChatGPT-4o) (Samuel et al., 2025). These parallel efforts demonstrate growing demand for agricultural AI and diverse methodological approaches to address farming challenges globally. AgriLLM, developed to handle farmer queries in India, focuses on approximately 4 million queries from Tamil Nadu across various sectors, including seasonal crops and diverse query types (Didwania et al., 2024). Agricultural AI models employ sophisticated training methods balancing general language skills with specialized knowledge. AgriGPT uses LoRA (Low-Rank Adaptation) for continual pretraining on agricultural literature, learning terminology while preserving broader capabilities (Yang et al., 2025b). Its data engine compiles comprehensive QA pairs across agriculture domains. CGIAR’s AgriLLM development involves stakeholder workshops where farmers, extension agents, and policymakers collaboratively generate realistic questions and evidence-based answers with citations (CGIAR, 2025). This human-in-the-loop approach ensures models learn agricultural facts alongside appropriate reasoning patterns and communication styles for different user groups.

## 5 Crop-specific LLMs: SeedLLM·Rice and other domain models

In recent years, agricultural AI has entered a new phase characterised by the emergence of crop-specific LLMs. Whereas generic foundation models (GFM), a specialised system, are trained on narrowly defined scientific bases, structured biological databases, and domain ontologies, allowing them to deliver contextually grounded, system-relevant, and agronomically relevant outputs. Such studies are particularly valuable for crops like rice, where decades of molecular research, breeding data, and field experimentation can be systematically encoded into AI systems.

### 5.1 Model architecture and training corpus

A practical example of this new paradigm is SeedLLM·Rice, a 7-billion-parameter transformer model trained on approximately 1.4 million rice-specific publications, representing almost complete coverage of the peer-reviewed scientific literature on *Oryza sativa* (Yang et al., 2025a). It was developed through a collaboration primarily involving the Yazhouwan National Laboratory (in Hainan), the Shanghai Artificial Intelligence Laboratory, and China Agricultural University. The feature of SeedLLM·Rice is its tight integration with a Rice Biological Knowledge Graph (RBKG) that incorporates Genome annotations (including the *Nipponbare* reference genome), Gene–trait associations, Transcriptomic and proteomic profiles compiled from more than 1,800 experimental studies, and Regulatory and metabolic pathway data (Yang et al., 2025a). This architecture enables models to be used across both unstructured text (e.g., scientific articles and reports) and structured biological entities, including genes, proteins, pathways, and traits. As a result, SeedLLM·Rice can infer gene–trait relationships, propose regulatory mechanisms, and generate biologically plausible hypotheses for experimental validation.

Another example is IPM-AgriGPT, a specialised LLM designed for integrated pest and disease management. This model utilises a Generation Evaluation Adversarial framework to construct a high-quality, domain-specific question-answer dataset. Further, it is optimised using Low-Rank Adaptation (LoRA) and Agricultural Chain-of-Thought Distillation (ACR-CoTD), which enables the transfer of complex expert reasoning patterns from a teacher model to a compact student model with reduced computational cost (Zhao et al., 2024).

### 5.2 Comparison with generic LLMs (e.g., GPT-4 / GPT-4o)

Crop-specific LLMs consistently outperform generic models on highly specialised tasks:

- Performance gains: According to evaluation studies, SeedLLM·Rice outperformed models like GPT-4o in rice biology challenges, achieving human-preferred results, ranging from 57 to 88%, mainly depending on the query complexity (Yang et al., 2025a).
- Improved factual accuracy: Since SeedLLM·Rice grounds its responses in the RBKG, there is a very low possibility of hallucinations or biologically implausible statements compared to general LLMs, which lack tight integration with structured molecular data (Yang et al., 2025a).
- Efficient and safe specialisation: IPM-AgriGPT’s use of LoRA means only a tiny part of model parameters are updated during fine-tuning, reducing computational cost. The G–EA framework ensures that its QA data is highly relevant, and the chain-of-thought distillation helps the model internalise domain reasoning patterns instead of relying on superficial memorisation (Yang et al., 2025a).

These advantages, however, come at a cost: developing such specialised models requires curated corpora, domain expertise, and continuous validation, especially for high-stakes agronomic advice.

### 5.3 Use cases in Rice agronomy, breeding, and pest management

There are several use cases of AI Models and their applications across crop domains (Table 1)

- Accelerating research: SeedLLM·Rice can help scientists generate hypotheses from extensive literature. The model can leverage both textual evidence and graph-based omics data to identify candidate genes (Yang et al., 2025a).
- Breeding strategy: By leveraging the RBKG, the model can prioritise candidate genes for crossbreeding, marker-assisted selection, or gene editing based on functional importance and expression profiles (Yang et al., 2025b).
- Trait mapping: SeedLLM·Rice supports genotype–phenotype inference due to its multi-modal reasoning, helping breeders map complex traits, such as yield under heat stress, to regulatory networks or expression signatures.
- Interactive advisory: IPM-AgriGPT can answer real-world pest management questions. The model synthesises domain-specific QA knowledge to propose social, cultural, biological, and chemical interventions (Zhang et al., 2025).
- Reasoned and safer recommendations: Using chain-of-thought reasoning, IPM-AgriGPT can weigh multiple strategies, acknowledging trade-offs, rather than recommending a single “best” solution (Zhao et al., 2024).
- Diagnostic support: When coupled with a knowledge retrieval system or a domain KG, IPM-AgriGPT can assist extension agents or agronomists in diagnosing pest or disease issues by linking symptoms to known biology and recommending plausible control measures (Zhang et al., 2025).
- KG + LLM disease systems: Emerging systems combine LLMs with knowledge graphs and neural networks to support disease diagnostics. For instance, expert systems for rice pests and diseases integrate structured knowledge and generative reasoning to interpret symptoms and suggest effective management strategies (Chen and Guo, 2025).
- Multimodal assistants, such as Agri-LLaVA, fuse vision and language—they are trained on datasets covering hundreds of pests and diseases. They can reason over both images and text, making them ideal for visual diagnosis in the field (Wang et al., 2024).
- Open-source plant science models: Foundational LLMs trained on large plant science corpora, such as PLLaMa-based models, provide a flexible base for creating crop- or domain-specific assistants (Gauba et al., 2026).

**Table 1:** Overview of AI models and their applications across crop domains.

Model	Domain/crop	Core architecture	Key innovation	Primary application	Evaluation status	Reference
SeedLLM·Rice	Rice biology and genetics	7B Transformer + Rice biological knowledge graph	Literature + omics + KG integration	Gene annotation, phenotype inference, hypothesis generation	Experimental/benchmark	Yang et al. (2025a)
IPM-AgriGPT	Pest and disease management	LLM fine-tuned with LoRA + G–EA	Adversarial QA + Chain-of-thought distillation	Integrated pest/disease advisory	Experimental	Zhang et al. (2025)
Agri-LLaVA	Multicrop diagnostics	Vision–language transformer	Image + text fusion	Visual diagnosis and advisory	Benchmark-only	Wang et al. (2024)
PLLaMa-Agro	Plant science	Domain-tuned LLM	Large plant science corpus	Foundation for crop LLMs	Experimental	Gauba et al. (2026)
Rice expert KG + LLM	Rice pathology	LLM + Knowledge graph	Structured reasoning on diseases	Disease diagnosis and control	Experimental	Wu et al. (2025)

Although crop-specific models demonstrate superior performance on highly specialized benchmarks, their advantages should be interpreted in the context of deployment requirements and use-case specificity. Models such as SeedLLM-Rice are optimized for rice biology, genetics, and agronomic reasoning, making them particularly valuable for research support, breeding programs, and crop-specific advisory systems. However, many practical extension applications involve broader questions spanning weather, markets, soil management, policy, and mixed-farming systems where general foundation models may offer greater flexibility. Furthermore, crop-specific models require extensive domain-specific corpus construction, expert curation, and periodic updating. Consequently, their benefits may not always justify the development cost when similar performance can be achieved through retrieval augmentation or domain-specific prompting of general foundation models.

### 5.4 Implications, risks, and future directions

Crop-specific LLMs can significantly expand access to specialised agronomic and biological parameters. They support decision-making among breeders, researchers, extension professionals, and farmers by translating complex scientific information into practical insights. These models can synthesise large volumes of literature and crop system data to accelerate hypothesis generation, improve breeding efficiency, and strengthen field-level management and recommendations (Kamilaris and Prenafeta-Boldú, 2018). Crop-specific LLMs can function as virtual agronomists when integrated into digital advisory platforms and offer specific guidance on crop nutrition, irrigation scheduling, pest and disease management, and varietal selection. This is mainly valuable in regions with limited access to extension services, where context-aware recommendations can enhance productivity and climate resilience (Sodano, 2024).

However, these applications also pose many challenges. The Model outputs require precise and robust validation by domain experts with authentic data to prevent inaccurate recommendations, particularly in sensitive parameters such as pesticide use or genetic guidance (Bommasani et al., 2022). Continuous model updating is necessary as scientific understanding, requirements and environmental conditions demand long-term institutional engagement and technical support. Accessibility remains a challenge for many smallholder farmers operating in multilingual and low-bandwidth environments. Without inclusive design, LLM-based solutions risk widening digital inequalities (Rustagi et al., 2024). Ethical concerns, including data ownership, transparency, user consent, and accountability, must therefore be addressed through clear governance frameworks and participatory system design (Bommasani et al., 2022). Future research must prioritise robust validation, multilingual optimisation, edge-based deployment, explainable AI, and integration with real-time climate and field data to ensure safe, equitable, and effective adoption.

### 5.5 Comparative analysis and deployment trade-offs

Although crop-specific LLMs such as SeedLLM-Rice demonstrate clear advantages in specialized domains, their deployment should not be interpreted as universally superior to general foundation models. Crop-specific models are most useful when the task requires deep biological, agronomic, or crop-specific reasoning, such as gene–trait inference, rice disease diagnosis, pest management, or localized advisory generation. However, these models require large, curated corpora, expert annotation, computational infrastructure, and continuous validation. Their benefits may therefore be limited in low-resource countries where access to GPUs, cloud infrastructure, high-quality datasets, and technical expertise is uneven. In contrast, general foundation models may be more scalable for broad advisory functions, especially when combined with domain-specific prompting, retrieval-augmented generation, or curated knowledge bases.

Fine-tuning, RAG, and KG-grounded architectures also involve different trade-offs. Fine-tuning can improve domain performance but is computationally expensive, difficult to update, and vulnerable to overfitting when trained on narrow crop-specific corpora (Table 2). This can reduce robustness when models encounter new agroecologies, emerging pests, climate anomalies, or cross-crop questions. RAG systems are more flexible because knowledge sources can be updated without retraining the model, but their reliability depends on retrieval quality, source credibility, and document coverage. KG-grounded systems offer stronger explainability, provenance, and structured reasoning, which are valuable for high-stakes advisory decisions, but they require substantial investment in ontology construction, multilingual standardization, expert validation, and long-term maintenance.

**Table 2:** Comparative analysis: crop-specific LLMs, general foundation models, fine-tuning, RAG, and KG-grounded architectures.

S. no.	Approach	Main advantage	Main limitation	Scalability	Computation cost	Best use
1	Crop-specific LLM	Strong domain accuracy	Narrow generalization	Low–medium	High	Rice biology, pest/disease advisory
2	General foundation model	Broad reasoning and flexibility	Weak crop-specific grounding	High	Low–medium	General advisory, multilingual Q&A
3	Fine-tuning	Internalizes crop knowledge	Overfitting and retraining burden	Medium	High	Stable domain tasks
4	RAG	Easy knowledge updating	Depends on retrieval quality	High	Medium	Dynamic advisory and extension
5	KG-grounded LLM	Traceable, explainable reasoning	KG construction and maintenance burden	Medium	Medium–high	High-stakes agronomic decisions

Therefore, crop-specific LLMs should be viewed as one option within a broader design space rather than as a default solution. For low-resource and smallholder contexts, hybrid systems combining general foundation models with RAG, curated local knowledge bases, and human expert oversight may offer a more sustainable pathway than fully retraining separate crop-specific models for every crop, region, or language.

### 5.6 Cross-crop LLMs

The approaches examined here are not, in principle, restricted to rice. Several crop-adapted generative models have emerged for broader use, including AgroLLM, AgriGPT, ShiziShanGPT, and the multimodal AgriLLaVA. In practice, however, these are either rice-focused, as with SeedLLM-Rice, or crop-general rather than tuned to a specific non-rice crop, and genuinely crop-specific models for staples such as wheat or maize remain scarce. Coverage across crops is therefore markedly uneven, concentrating on a few high-value cereals while most regionally important crops have little dedicated development. A natural extension is generative-AI-supported comparative analysis across crops, including comparative genomics and cross-crop trait inference. The data and reasoning building blocks exist, but we found no study that operationalises such cross-crop comparison and validates it against genomic or agronomic ground truth. We flag this as a promising but presently speculative direction, dependent on shared, interoperable multi-crop resources that do not yet exist at scale.

## 6 Enhancing LLM utility through knowledge graphs

### 6.1 Agricultural knowledge graphs

Agricultural knowledge graphs (KGs) are structured networks of agricultural entities, including crop varieties, traits, pests, genes, soil types, and their relationships. By encoding domain-specific knowledge in a graph, KGs enable the storage, querying, and reasoning over complex, multi-relational information in a machine-based, interpretable format (Wang and Shi, 2025). These graphs present as semantic backbones that support fact-based decision-making and mitigate the risk of misinformation when LLMs are utilised in agriculture.

In agriculture, KGs often leverage controlled vocabularies and ontologies such as AGROVOC or the Crop Ontology to standardise terms and ensure consistency across different datasets and platforms (Wang and Shi, 2025). In national contexts such as India, these KGs can be extended with local data (variety-release records, soil health data, and regional trial metadata) to power context-aware AI systems that understand and reason about local conditions.

### 6.2 Integration methods: embeddings and retrieval-augmented reasoning

To make knowledge graphs useful for LLMs, researchers use sophisticated integration strategies that combine structured graph reasoning with unstructured language generation.

1. Embedding-Based Integration: Graph embeddings (e.g., TransE, node2vec, or Graph Neural Networks) project KG nodes and relationships into a continuous vector space. These embeddings align with text embeddings, enabling joint retrieval across both structured and unstructured data. When a user query comes in, both the KG embeddings and document embeddings are searched. The LLM then uses the retrieved graph substructure and texts to generate responses grounded in structured knowledge.
2. Retrieval-Augmented Generation (RAG) with KG: In KG-RAG, the LLM is augmented not only with retrieved text but also with factual triples from the KG. This setup enhances accuracy, particularly for multi-hop or reasoning-intensive queries, by incorporating explicit relational knowledge (Linders and Tomczak, 2025). More advanced systems decompose complex queries into sub-questions (using chain-of-thought reasoning), retrieve relevant graph paths, and then synthesise the answer. This enhances explainability because the system can trace which graph edges supported each part of the answer (Linders and Tomczak, 2025).
3. Hybrid Symbolic–Neural Pipelines: In hybrid systems, a symbolic component (e.g., SPARQL query engine) extracts precise facts or subgraphs from the KG, while a neural LLM synthesises this information into human-readable text. This architecture offers both auditable reasoning and conversational fluidity (via the LLM), making it well-suited for sensitive domains such as agriculture (Zhao et al., 2024).

### 6.3 Applications: varietal recommendation and trait inference

Integrating KGs with LLMs unlocks powerful use cases in agricultural decision support, especially in breeding and extension:

- Varietal Recommendation: By encoding data on crop varieties, local soil properties, climate zones, and yield trial results into a graph, a KG + LLM system can generate contextual recommendations for which variety to plant. The system can explain its choice by tracing relevant KG paths (e.g., variety → yield in similar agro-ecology → disease resistance) and generating user-friendly advice rooted in data.
- Trait Inference and Breeding Support: In breeding, a KG that includes genomic annotations, marker-trait associations, (quantitative trait locus) QTLs, and environmental interactions can help the LLM infer likely candidate genes for traits. The LLM can generate hypotheses based on graph substructures, supporting breeders with scientifically grounded insight into which genes to prioritise in crossbreeding or marker development.
- Pest & Disease Diagnosis: A KG + LLM framework can map symptom descriptions to pest or disease entities, suggest causal relationships (e.g., *disease → pathogen → management strategy*), and reason over recommended interventions using the graph’s structured knowledge (Zhao et al., 2024).

### 6.4 Challenges: ontology, data, and multilingualism

While the integration of KGs and LLMs shows substantial promise for digital agricultural advisory, several persistent challenges limit real-world implementation, particularly in the Indian context.

#### 6.4.1 Ontology construction

The construction of robust agricultural ontologies is constrained by substantial regional variability in terminology of the diseases and pest names, crop variety naming, measurement conventions, and management practices. In India, vernacular crop names and location-specific agronomic traits introduce semantic inconsistencies that hinder standardisation and machine interpretability. Studies have shown that incomplete or poorly aligned ontologies significantly degrade KG performance and reasoning accuracy (Bhuyan et al., 2021; Xhani et al., 2025). Although global vocabularies such as AGROVOC provide a strong semantic backbone, they often lack sufficient local granularity for national-scale applications and require extensive contextual adaptation (Xhani et al., 2025).

#### 6.4.2 Data gaps and interoperability

A key barrier is the fragmentation and inaccessibility of agronomic datasets, especially those derived from smallholder field trials, local varietal evaluations, and fine-resolution soil surveys. These data are frequently stored in unstructured formats, lack standard metadata, and are distributed across disconnected institutional silos (Wolfert et al., 2017). The lack of semantic interoperability across data platforms further complicates KG construction. Heterogeneous formats and inconsistent data models increase manual pre-processing requirements and introduce integration errors (Kamilaris et al., 2017; Wilkinson et al., 2016).

#### 6.4.3 Multilingual representation

The multilingual representation remains a critical limitation in agricultural KGs. As India’s linguistic diversity across states has its own languages, most existing ontologies and KGs are designed primarily for English-language environments. This restricts usability for farmers and extension personnel who operate in regional languages. Research shows that language bias in knowledge infrastructures significantly reduces knowledge accessibility and trust among local users (Bender et al., 2021). While multilingual thesauri exist, scalable integration of multilingual entities, relations, and synonyms into operational KGs remains technically immature (Xhani et al., 2025).

#### 6.4.4 Maintenance and governance

Agricultural knowledge evolves rapidly due to new varieties, shifting pest and disease pressures, and climate-driven changes in management practices. Maintaining a KG, therefore, requires robust governance structures, provenance tracking, and versioning frameworks. A lack of clear institutional ownership of knowledge infrastructure has been identified as a significant risk to long-term sustainability. Provenance-aware KGs are also essential to ensure trust, transparency, and scientific reproducibility.

Although KG-grounded architectures improve explainability and traceability, they introduce substantial operational challenges. Constructing and maintaining agricultural knowledge graphs requires expert involvement, continuous ontology updates, provenance tracking, and integration of heterogeneous datasets. For rapidly evolving domains such as crop protection and climate adaptation, maintaining current knowledge graphs may become as resource intensive as maintaining domain-specific language models themselves. These hidden maintenance costs are rarely considered in benchmark evaluations but represent an important factor in long-term deployment sustainability.

## 7 Multimodal sensing for smart rice advisory

Innovative advisory systems primarily rely on multimodal sensing, integrating data streams from UAVs, satellites, and smartphones to support timely, location-specific, and evidence-based decision-making. In Indian rice-based systems, where spatial variability, climatic uncertainty, and smallholder dominance significantly influence production outcomes, multimodal sensing offers a transformative pathway to enhance resilience and sustainability (Barbedo, 2022). Although multimodal sensing is advancing rapidly, its large-scale operational use in rice advisory systems remains limited. A central constraint is the technical complexity of fusing heterogeneous data sources, as satellite and UAV datasets differ markedly in spatial, temporal, and spectral resolutions, requiring high computational capacity for real-time integration (Zhang, 2010). The limited adoption of interoperable data standards further restricts seamless system integration. In many rice-growing regions, especially smallholder-dominated landscapes, infrastructure bottlenecks, including unreliable rural connectivity, limited access to cloud-based computing, and the high cost of advanced sensors, constrain scalability (Barbedo, 2022). The scarcity of high-quality, spatially diverse ground-truth datasets, particularly from rainfed, flood-prone, and saline rice ecologies, reduces the robustness and cross-regional transferability of AI-driven advisory models (Allu and Mesapam, 2026). Institutional fragmentation among agricultural research bodies, meteorological agencies, irrigation authorities, and extension systems, combined with weak data-sharing mechanisms, further limits system-level integration and national-level scaling.

### 7.1 Drone, satellite and smartphone imaging

Satellite and UAV-acquired data now form the backbone of operational precision crop monitoring. Platforms such as Sentinel-2 and Landsat enable consistent, wide-area observation of crop vigour, growth dynamics, and seasonal variability through vegetation indices, including NDVI and EVI (Atzberger, 2013). In contrast, UAVs provide ultra-high-resolution observations of canopy structure, biomass distribution, water stress and nutrient dynamics, allowing the detection of subtle crop stress patterns that are not easily captured from space (Xu et al., 2021). The integration of multispectral, hyperspectral, and thermal sensors on UAVs enhances the capacity to monitor crop health during key phenological stages (Sahoo et al., 2025). These time-series measurements are crucial for rice, as rapid growth cycles necessitate timely interventions to ensure optimal yields. Simultaneously, smartphones have emerged as a powerful tool for participatory sensing. Farmers can capture geotagged images of symptomatic leaves or crop damage, which AI-powered applications can process to identify diseases or nutrient deficiencies. With increasing smartphone penetration in rural India (79.2% of males and 75.6% of females aged 15 and above), mobile imaging serves as a low-cost, scalable mechanism for connecting farmers to real-time advisory ecosystems (Kamilaris and Prenafeta-Boldú, 2018).

### 7.2 Disease detection and phenotyping using AI

AI, based on Deep Learning and computer vision, has significantly advanced rice system disease detection. CNN have been demonstrated to have high accuracy in identifying major rice diseases such as blast, bacterial blight, and sheath rot from leaf images (Mohanty et al., 2016). These capabilities are now being transitioned to real-world settings through mobile and cloud-based platforms. Beyond disease diagnosis, AI enables high-throughput phenotyping by extracting structural and physiological traits such as canopy cover, plant height, tiller density, and stress response from time-series imagery (Yang et al., 2017). When combined with UAV and satellite data, these systems allow researchers and extension agencies to assess varietal performance and monitor crop responses to changing climatic conditions. In a country like India, where microclimatic and soil variability significantly influence rice growth, AI-based phenotyping can support location-specific varietal recommendations and adaptive management strategies, thereby reducing both yield gaps and input inefficiencies.

### 7.3 Vision–language fusion in recommendation systems

While image-based detection represents a significant technological advancement, farmers ultimately require clear and actionable guidance. Vision–language fusion models are designed to bridge this gap by converting visual detection outputs into natural-language advisory messages. Such systems improve both interpretability and adoption, especially for smallholder farmers. When paired with regional language translation, they address critical linguistic barriers in multilingual agricultural contexts such as India (Jiang et al., 2025). Plantix is an AI-powered mobile app developed by PEAT GmbH that acts as a crop doctor, diagnosing pests, diseases, and nutrient deficiencies from user-uploaded photos across 30 + major crops. It supports 780 + plant issues, provides treatment recommendations (chemical or natural), fertiliser calculators, weather forecasts, and disease alerts in 19–20 languages (Plantix, 2017). Although still in its early stages for rice systems, vision–language fusion represents an essential step toward explainable and farmer-friendly AI, moving from detection to decision support.

### 7.4 Role of citizen science and farmer feedback

The data generated through Citizen Science is central to strengthening multimodal advisory systems. Farmers contributing field images, geotagged observations, and management outcomes create a distributed monitoring network that enhances model training, validation, and continuous improvement (Fritz et al., 2019). This participatory feedback loop does more than improve algorithms; it builds trust, ownership, and digital inclusion. When linked with national platforms such as India’s Digital Agriculture Mission and AgriStack, citizen science-driven data can inform regional disease surveillance, early warning systems, and policy-level planning (GoI, 2021). However, challenges related to data privacy, digital literacy, gender equity, and access disparities must be addressed through transparent governance frameworks and farmer-centric policies.

### 7.5 Data, science and policy gaps

While multimodal sensing technology has advanced rapidly, its adoption in rice advisory systems faces major obstacles. The biggest challenge is the lack of quality, well labeled datasets from diverse growing environments, especially rainfed, flood prone, and marginal zones most vulnerable to climate shifts. These underrepresented regions limit model accuracy across different landscapes (Charles et al., 2010; Kamilaris and Prenafeta-Boldú, 2018). Current AI systems also lack integration with crop models that account for soil plant atmosphere dynamics and climate stress, reducing reliability (Kamilaris and Prenafeta-Boldú, 2018). Additionally, models trained on controlled conditions struggle in variable smallholder farms (Kamilaris and Prenafeta-Boldú, 2018). Policy gaps including absent validation standards, weak links to extension services, and unresolved issues around data ownership and algorithmic transparency further hinder adoption (Bommasani et al., 2022). Progress requires region specific datasets, open data infrastructure, and ethical frameworks ensuring farmer centered deployment.

### 7.6 Multimodal sensing for smart rice advisory for small holder farmers

The data pipelines that ground these systems already work in the field. Crop simulation models such as DSSAT CERES Rice integrate weather, soil, and cultivar information to predict yield and quantify stress, while UAV based phenotyping reads crop condition directly from the canopy. Khose and Mailapalli (2024) paired UAV multispectral imagery with random forest regression to predict rice nitrogen status (SPAD) with R^2^ up to 0.91, producing maps that pinpoint where stress sits within a plot. The same approach extends to drought tolerance, a trait that matters more each year as climate change intensifies water stress in rice. When rice plants run short of water they respond in visible ways such as shorter stature, rolled leaves, warmer canopies, fading chlorophyll, and sterile spikelets, and high throughput phenomics platforms can capture these signs across thousands of plants far faster than anyone scoring them by hand (Anand et al., 2025). What these tools share is a limitation. They produce technically precise outputs that only trained agronomists can configure and interpret, which keeps them out of reach of the smallholder farmers who most need them. This is the gap generative AI can close. A large language model could sit on top of these validated pipelines as a natural language interface, taking an everyday question, pulling the relevant model output or sensing data, and answering in the farmer’s own language with advice that fits their specific field and season.

The value becomes clear in the kinds of exchanges such a system could support. A farmer might ask, “My transplanting got delayed this year, will the monsoon rain be enough for my crop?” Rather than returning a yield curve, the tool could draw on a calibrated crop model and the local rainfall forecast to reply that the late start narrows the safe window, and that choosing a shorter duration variety or arranging supplementary irrigation at flowering would protect the harvest. Another farmer photographing yellowing leaves could ask, “Why is my rice turning pale and what should I do?” The system could compare the image against nitrogen status patterns of the kind mapped by Khose and Mailapalli (2024), recognise the signature of nitrogen deficiency rather than disease, and recommend a specific top dressing of urea timed to the crop stage.

The same interface could turn research findings into timely warnings. Because Jha et al. (2020) showed that water stress during the reproductive stage can cut rice yield by 43%, a farmer asking, “It has not rained for a week, is my crop in danger?” could be told that the field is entering its most sensitive phase and that irrigating within the next few days is the single most important step they can take this season. A farmer planning ahead might ask, “Which variety should I plant next season on my plot?” and receive a suggestion matched to their soil type, local climate record, and the drought signals captured by high throughput phenotyping, rather than a generic recommendation. Even broad questions such as “Will prices be good if I plant rice this year?” could be partly informed by regional production forecasts of the kind generated from the SAR assimilated yield maps of Pazhanivelan et al. (2022), helping the farmer weigh the decision with information that was previously available only to planners. These examples come from only a few studies, yet they hint at a much larger capability. The deeper promise of generative AI and large language models is their ability to translate the enormous and growing body of published agricultural science into clear, actionable advice that reaches the smallholder farmer directly.

For India’s smallholders, this combination is what makes automated advisory realistic at scale. A farmer with a basic phone could hold this kind of conversation in a regional language, at the exact moment a decision needs to be made, without an extension agent physically present. Crucially, the crop model and phenotyping layer keep the system honest. They supply the factual grounding that constrains what the language model says, reducing hallucination and anchoring every recommendation in validated agronomic science rather than plausible sounding guesswork.

## 8 Integrating LLMs with crop simulation models

### 8.1 DSSAT, ORYZA, and APSIM as domain simulators

Process-based crop simulation models have been central to agricultural decision-making for more than forty years. Among these, the Decision Support System for Agrotechnology Transfer (DSSAT) is widely used. It uses a daily time step and integrates modules for photosynthesis, phenology, water balance, and nitrogen dynamics, covering more than 42 crops, including rice (Jones et al., 2003; Hoogenboom et al., 2019; Abayechaw, 2021; Gavasso-Rita et al., 2024). These mechanistic models capture how crops grow and develop by mimicking the physiological and biophysical processes that shape plant responses to weather, management, and genetic traits. ORYZA (v3) provides rice-specific modelling with highly detailed representations, including ORYZA2000 for potential production analysis, ORYZA_N for nitrogen dynamics, and ORYZA_PSP for pest and disease impacts (Bouman et al., 2001; Li et al., 2017). The model has been extensively validated across Asian rice production systems, demonstrating robust performance in simulating yield responses to water management, nitrogen application, and varietal differences under diverse environmental conditions (Bouman and Van Laar, 2006).

The Agricultural Production Systems sIMulator (APSIM) is a comprehensive biophysical model developed in 1991 by CSIRO and Queensland State Government agencies to simulate agricultural systems and their responses to climate, soil, genotype, and management interactions (Keating et al., 2003). The framework is structured around plant, soil and management modules, including a diverse range of crops, pastures and trees, soil processes including water balance, nitrogen and phosphorus transformations, soil pH, erosion and a full range of management controls APSIM (Holzworth et al., 2014). Operating on a daily time-step basis using meteorological data and soil characteristics, APSIM predicts crop phenology, biomass accumulation, yield, and resource use efficiency across varying conditions (Tan et al., 2024). The model has been validated globally across diverse cropping systems, demonstrating reliable simulation capabilities (APSIM, 2024). Its modular design supports multiple applications including on-farm decision-making, sustainable cropping system design, climate change impact assessment, and optimization of nutrient and water management, making it invaluable for climate adaptation strategies and agricultural sustainability research (Keating et al., 2003).

These simulation platforms offer key advantages for agricultural decision-making by mechanistically modelling interactions among water, nutrients, and genetics, enabling projections beyond existing data, which is vital for climate change adaptation (Asseng et al., 2015). They have shown reliability through validation across diverse agroecological zones (Raymundo et al., 2017). However, adoption barriers include complex parameterization, for example, over 40 parameters in DSSAT covering planting, cultivars, fertilizers, and soils (Jones et al., 2003). Their use demands specialized skills for calibration, output interpretation, and translating results to practical advice, limiting use mostly to research and advanced consulting (Rosenzweig et al., 2013). Additionally, static embedded knowledge requires developer updates, leading to delays in integrating new scientific findings into tools (Keating et al., 2003).

### 8.2 LLMs for configuring, interpreting, and communicating simulation outputs

The integration of LLMs with crop simulation models represents a transformative approach to agricultural decision support systems. This workflow demonstrates how natural language interfaces can democratise access to sophisticated agronomic modelling tools such as DSSAT, ORYZA, and APSIM. Farmers submit natural-language questions at the start of the process, which the LLM model analyses to identify key factors such as crop type, season, location, and relevant agricultural conditions. For over 30 years, DSSAT, a widely used crop modelling system, has been used worldwide to simulate crop growth and evaluate management decisions (Jones et al., 2003). The natural language question is then transformed by the big language model into structured inputs for the simulation model, which include cultivar characteristics, soil profiles, meteorological information, and management techniques including irrigation schedules, fertilizer application, and sowing dates. After the inputs are configured, crop simulation models generate raw outputs on daily growth, water and nutrient dynamics, and stress indices for heat, drought, and flooding. APSIM provides a robust framework for simulating crop and soil biophysical processes under varied environmental conditions (Holzworth et al., 2014). Since these outputs are complex and data-heavy, large language models play a crucial role in translating them into clear and usable insights for end users.

**Figure 4:**
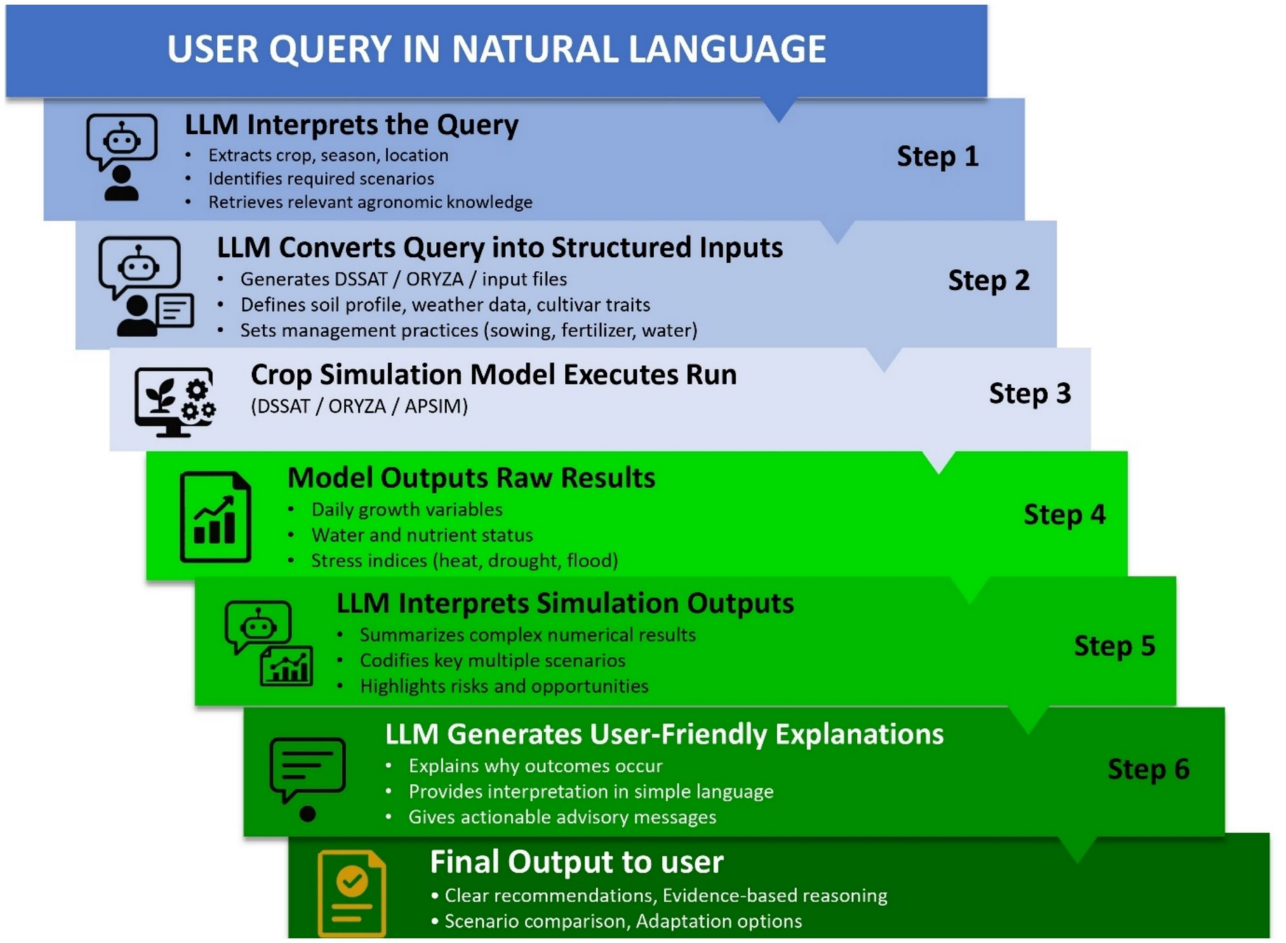
Workflow of LLM-based agricultural decision support system.

In the final stages, the LLM synthesizes simulation outputs into comprehensible summaries, highlighting risks and opportunities while codifying results across multiple scenarios (Figure 4). Recent advances in natural language processing have enabled the development of intelligent agricultural advisory systems that can translate complex model outputs into actionable recommendations for farmers (Sharma, 2021). This human-centred approach bridges the gap between computational agronomy and practical farm-level decision-making, enabling farmers to leverage advanced modelling tools without requiring specialized technical expertise.

**Figure 5:**
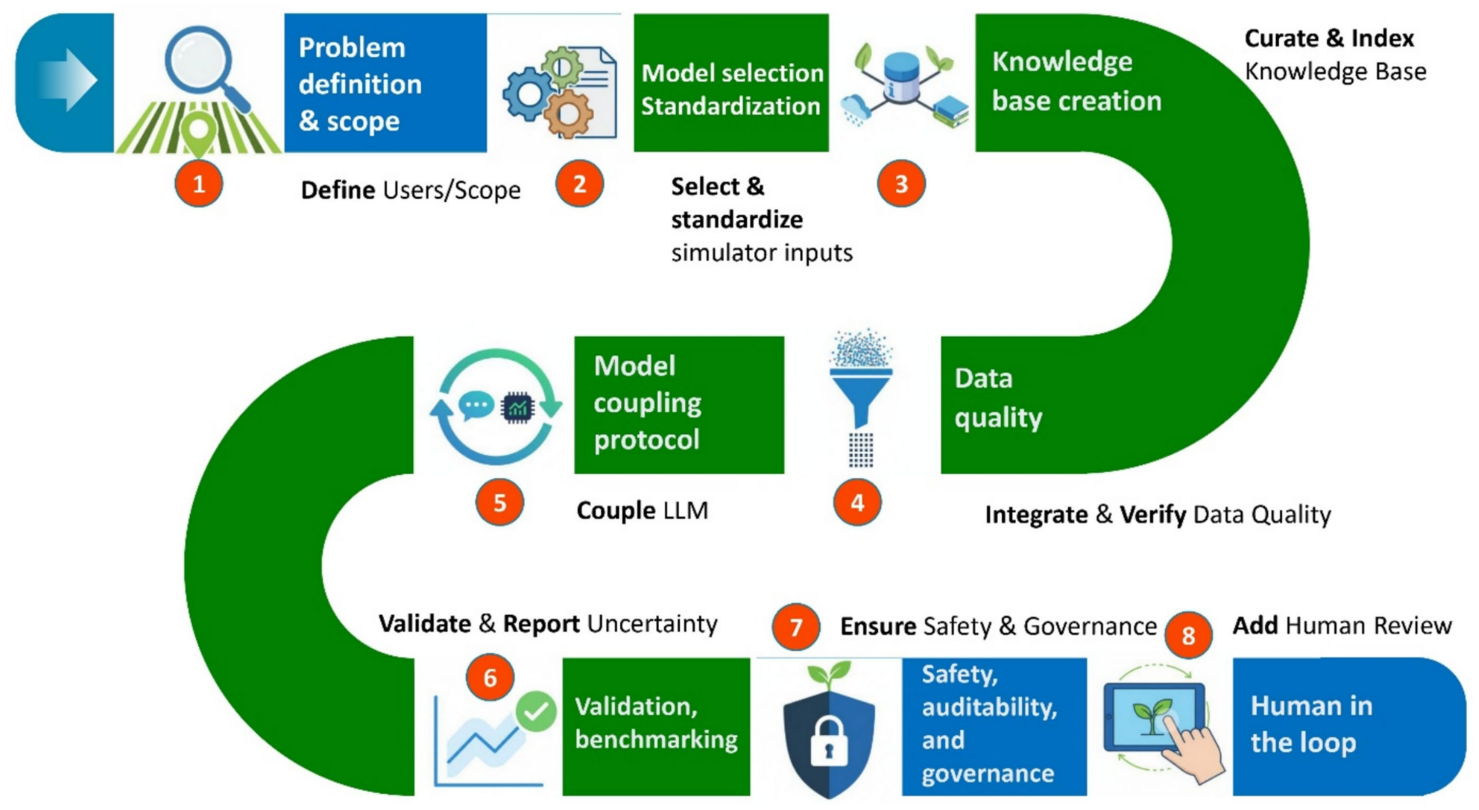
Comprehensive framework for data, model integration, and governance in LLM-driven simulations.

Successfully integrating LLMs with crop simulation models for real-world agricultural decision making requires a development framework that genuinely prioritises user trust, scientific soundness, and fair access. The eight-stage framework is shown in Figure 5. provides a clear roadmap for building such systems responsibly, from the earliest design steps through deployment, capturing the best practices emerging in today’s agricultural AI landscape.

#### 8.2.1 Stage 1: problem definition and scope

The development process begins by identifying target farmer groups in regions with unique agroecologies and key crops that require decision support (Jones et al., 2017). Setting scope limits prevents feature creep and keeps focus on specific agricultural challenges. For example, a system targeting rice farmers in Punjab must specify if it addresses only irrigated systems or also rainfed environments, which demand different models and data. Participatory approaches demonstrate that the system effectively addresses farmers’ concerns, rather than merely promoting technology-based solutions (Steinke and van Etten, 2017).

#### 8.2.2 Stage 2: model selection and standardisation

Choosing crop simulation models requires balancing scientific rigor, data needs, and computational efficiency. Standardising model inputs following ICASA (International Consortium for Agricultural Systems Applications) standards ensures consistent formatting for weather, soils, and management data (White et al., 2013), enabling interoperability and easier maintenance. Regional calibration data such as genetic coefficients, soil properties, and historical weather capturing climatic variability are critical (Dzotsi et al., 2013).

#### 8.2.3 Stage 3: knowledge base creation

A wide-ranging knowledge base is essential for training and using LLMs. It brings together digitized extension resources, crop management studies, databases of crop varieties, pest and disease control guidelines, and information on available inputs (Zhai et al., 2020). Experts review the content to keep it accurate and relevant, and the data is organised for easy searching. In a multilingual country like India, supporting all 22 scheduled languages is crucial to making the information accessible to everyone (Birner et al., 2021). This knowledge base is continually updated with new research and feedback from farmers, growing in scope and relevance over time.

#### 8.2.4 Stage 4: data quality integration and verification

Data quality is crucial for ensuring accurate models and reliable recommendations. The integration process manages diverse data sources, including satellite images from Sentinel-2 and MODIS, weather station records, soil survey data, farmer-reported inputs, and market information (Beza et al., 2017). Quality control procedures identify anomalies, fill in missing data, and flag unrealistic values. Governance policies outline data ownership, access rights, and update schedules to maintain current and trustworthy systems.

#### 8.2.5 Stage 5: model coupling protocol

Robust LLM-model coupling needs clear interfaces and communication protocols. The architecture usually includes three layers: the conversational LLM interface that receives user queries, the integration layer that translates natural language into model inputs and interprets outputs, and the simulation layer that runs crop models and returns results (Piyathilake et al., 2024).

#### 8.2.6 Stage 6: validation and uncertainty reporting

Strong validation builds credibility through multi-location field trials comparing predictions to actual yields (Wallach et al., 2018). Validation data must remain unbiased. Sensitivity analysis identifies key traits affecting outputs, highlighting data quality issues. Systems communicate uncertainty through confidence labels. Benchmarking against existing advisory services tests if LLM-based systems outperform current extension advice (Basso and Liu, 2019). Post-deployment monitoring ensures performance maintains stability as farming conditions evolve.

#### 8.2.7 stage 7: Safety, auditability, and governance

Safety mechanisms prevent inadequate AI advice in agriculture. Rule based checks flag biologically implausible outputs, like excessive fertilizer or pesticides during flowering, routing them for expert review before sharing (Jakku et al., 2019). High stakes recommendations, pest chemicals, major investments, or irreversible actions require agronomist approval. The system logs user actions, inputs, simulations, and outputs for transparency, auditing errors while anonymizing personal data to protect privacy. Strong governance defines developer, expert, and operator roles. Terms of service clarify AI offers guidance without guarantees; users bear final responsibility (Dara et al., 2022). Oversight by farmers, agronomists, data scientists, and policymakers ensures ethics and practicality.

#### 8.2.8 Stage 8: human-in-the-loop integration

Human knowledge with automated tools improves agricultural decision-making. Extension officers and agronomists contextualize AI recommendations to local farming conditions, answer queries AI cannot handle, and demonstrate practical implementation methods (Gao et al., 2024). FarmerChat, an AI-powered conversational assistant developed by Digital Green for smallholder farmers, offers real-time, localized agricultural advice via text, voice, or images in local languages. It supports over 565,000 users across India, Kenya, Nigeria, Ethiopia, and Brazil, resolving more than 5 million queries on crop diagnosis, weather, pests, and markets (Digital Green, 2025). Training programs, farmer feedback loops, structured data collection, community validation, and human-in-the-loop approaches combine human knowledge with AI systems to create practical, culturally respectful agricultural solutions (Birner et al., 2021).

### 8.3 Framework implementation: iterative and adaptive

This framework has eight stages and works in a repeating, circular way rather than following a straight line. Results from testing the system can lead to improvements in the information it uses and the quality of its data. Feedback from farmers is collected regularly, helping to update knowledge and adjust the models to work better. The framework emphasizes flexible management, meaning the AI system must keep evolving as farming practices, climate conditions, and technologies change over time.

### 8.4 Validation framework and implementation considerations

**Figure 6:**
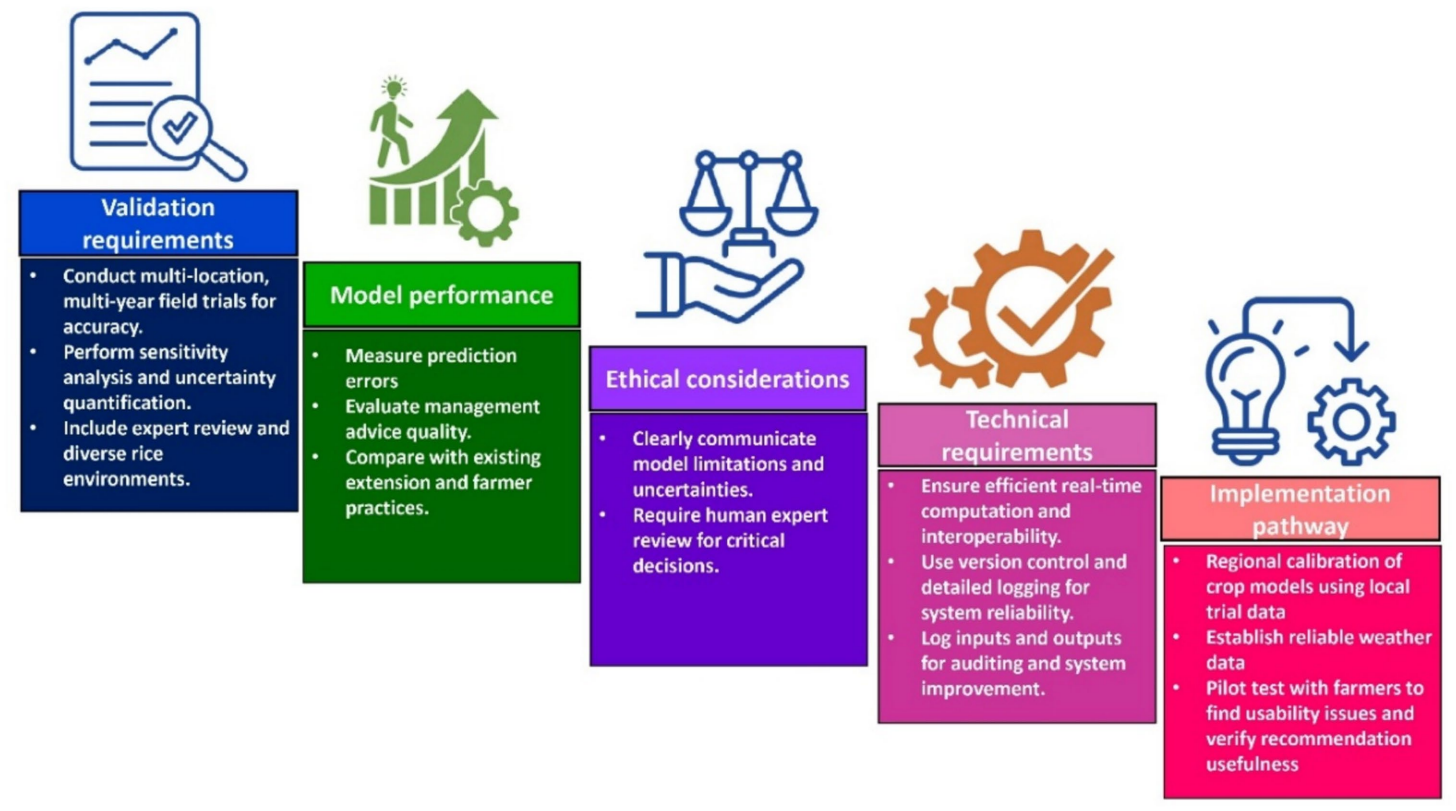
Framework for validation and implementation of LLM-aided decision support in agriculture.

Using LLMs and crop models together to support real-world farming decisions requires careful testing and strong attention to ethics. The successful deployment of LLM-crop model systems require rigorous validation in diverse environments, clear ethical guidelines, robust technical infrastructure, and participatory implementation with continuous refinement based on farmer input. This integration can transform generic agricultural advice into personalised, science-based guidance at scale but demands sustained investment in validation, capacity building, and equity considerations (Figure 6).

Validating crop models and AI-based agricultural systems requires careful, continuous testing to ensure they perform correctly under real-world conditions. Multi-location and multi-year field trials are essential to assess whether the models yield accurate results across different regions and climates. At the same time, sensitivity analysis helps confirm that the model’s parameters remain within reasonable ranges, and uncertainty quantification provides confidence in the predictions (Wallach et al., 2018). Before farmers use these systems, agronomic experts should carefully review the recommendations to ensure they are biologically sound and practical for field conditions (Jones et al., 2017). For rice systems, especially, validation should include a wide range of growing conditions, such as irrigated lowlands, rainfed uplands, and flood-prone zones, to ensure the models are robust across different production environments.

To evaluate model performance, both numerical accuracy and practical usefulness must be assessed. This includes analysing yield prediction errors using mean absolute error and root mean square error, as well as judging whether management suggestions are appropriate and realistic for actual farming contexts (Willmott and Matsuura, 2005). Comparing AI-based outputs with existing extension recommendations and farmer practices helps determine whether these tools offer meaningful improvements over current decision-support methods. Transparency about model limitations is crucial to ensure that users apply the system responsibly, since crop models simplify complex biological processes and always include some uncertainty (Lobell and Burke, 2010). Decisions involving high-risk actions, like pesticide use or significant investments, should constantly be reviewed by experienced human experts rather than relying solely on AI (Jakku et al., 2019). Liability frameworks must clearly define the roles of AI and agronomists, stating that AI outputs are advisory and not guaranteed results (Dara et al., 2022).

Accessibility for all farmers is vital to avoid inequality between digitally skilled and less literate users (Birner et al., 2021). Technical success depends on real-time responsiveness (Beza et al., 2017), well-structured APIs (Martre et al., 2015), and strong data systems for weather and crop varieties (Hansen et al., 2014). Farmer feedback and localized testing ensure continuous learning and improvement (Steinke and van Etten, 2017; van Klompenburg et al., 2020).

## 9 Current institutional and policy efforts in advancing AI applications in agriculture

India’s National AI Mission, launched in 2024, is a bold effort to establish India as a global leader in AI, with a special focus on agriculture. Some of the key initiatives of the Government of India (GoI) and Global Initiatives are provided in Tables 2, 3. It supports a wide range of programs, including investments in high-performance computing, development of AI models customised for Indian agriculture, and creation of unified digital farmer databases called Agri Stack (GoI, 2024). These initiatives enable personalised advisory services, improved access to credit, insurance automation, and targeted input subsidies (Table 4).

**Table 3:** Key programs and initiatives under India’s national AI mission and agricultural AI ecosystem.

Key program or initiative	Aim of the initiative	Budget /outlay	Year/timeframe	Institute/stakeholders	Reference
National AI Mission (India)	Position India as global AI hub; democratize AI access in sectors including agriculture	1,152 million USD	Launched 2024	NITI Aayog, IIT Madras, IISc Bangalore, ICAR, Microsoft AI for Earth, Google AI	IndiaAI, (2024) and Microsoft (2022)
AgriStack	Create unified farmer databases for personalized AI advisory	Not specified	2021	NITI Aayog, MoA and FW	IndiaAI (2024)
AI Pilot Projects (FASAL, Kisan Rath, eNAM)	Deploy AI for crop monitoring, logistics, market analytics	Not specified	2022–2023	MoA and FW, ISRO	MoA and FW (2022, 2023) and ISRO (2023)
MahaAgri-AI Policy (Maharashtra)	Pioneer agricultural AI adoption with training and funding support	56 million USD	Announced 2024	Government of Maharashtra, MRSAC, Mahatma Phule Krishi Vidyapeeth, Dr. Balasaheb Sawant Konkan Krishi Vidyapeeth	Government of Maharashtra (2024)
TIDE 2.0	Support electronics and IT startups with agri-tech applications	Not specified	2022	MeitY	MeitY (2022)
SATYAM	AI for marginal and smallholder farmers in rainfed regions	Not specified	Recent	MoA and FW	MoA and FW (2024)
SERB Grants	Research in climate-resilient agriculture, forecasting, nutrient management	5.6 million USD annually	Ongoing	DST, IIT Kanpur, IARI	DST (2023)
Atal Innovation Mission (AIM)	Support startups across sectors including agri-tech; innovation challenge	Not specified	Current	AIM	NITI Aayog (2023)
RKVY-RAFTAAR	Support agri-innovation projects; drone monitoring, AI chatbots, blockchain	112 million USD annually	2020–2024	MoA and FW	MoA and FW (2024)
Startup India Seed Fund	Early-stage venture funding for agritech startups	105 million USD	Current	DPIIT, NABARD, BIRAC	NABARD (2023); BIRAC (2022)

**Table 4:** Key global digital public good policies driving agricultural digitization.

Policy or initiative	Implementing agency/country	Type of digital public good	Agricultural application	Key features	Target beneficiaries	Year/period	Impact/expected outcomes
CGIAR Open Data and Open Tools	CGIAR (Global)	Open datasets, crop models (DSSAT, APSIM), geospatial layers	Climate smart advisories, crop modelling, risk management	FAIR data principles (Findable, accessible, interoperable, and reusable), open-source models, global accessibility	Researchers, extension agencies	2015-present	Improved research reproducibility and low-cost advisory innovation
Global Digital Public Goods Alliance (DPGA)	United Nation (Global)	DPG registry, open-source governance	Scaling digital agri-tools globally	Certification framework, community validation	Low and Middle-Income Countries (LMICs) innovators	2019-present	Global collaboration, quality assurance for digital public-good solutions
USDA Open Agricultural Data Initiative	United States	Open datasets, weather and soil data, research outputs	Crop modelling, risk prediction, agronomic planning	Climate and soil databases, public repositories	Farmers, start-ups, researchers	2018-present	Improved innovation and private-sector digital product development
FAO Digital Agriculture Strategy Framework	FAO (Global)	Open data standards, open-source tools for extension	Digital advisory, early warning, food security monitoring	Normative standards, open frameworks, e-extension tools	Governments, extension systems	2019-present	Strengthened national digital ecosystems and improved advisory reach
World Bank Agriculture Observatory	World Bank	Open geospatial DPGs, dashboards, APIs	Climate-smart planning, monitoring, insurance	Geospatial layers, crop maps, risk dashboards	Governments, development agencies	2021-present	Better evidence-based planning and climate risk management
Smart Africa Digital Agriculture Blueprint	African Union/Smart Africa Alliance	Open standards, open APIs, digital registries	Extension, digital markets, weather services	Interoperable digital ecosystems, farmer databases, innovation sandbox	African smallholders	2020–2030	Harmonized digital policies, scalable e-agriculture services

### 9.1 Digital public goods (DPI) and policy interventions

Digital Public Infrastructure (DPI) in agriculture is a government-led framework that provides open-source AI models, public datasets, and shared APIs to support innovation, data accessibility, and efficient service delivery for farmers, researchers, and social enterprises. India’s DPI promotes open, reusable AI tools to reduce duplication and treat agricultural AI as a public good (NITI Aayog, 2023; UNESCO, 2022). Policies ensure farmer data sovereignty, benefit sharing, and explainable AI advisories, while interoperability standards and bias audits prevent vendor lock-in and ensure fairness across crops, regions, and communities (FAO, 2019).

### 9.2 Role of public-private pilots

The Indian Council of Agricultural Research (ICAR) established a Virtual Centre for Excellence on AI in Agriculture to drive AI-based agricultural research and consolidate information on AI products and methodologies (ICAR-IASRI, 2025).

The Indian Space Research Organisation (ISRO) provides satellite data infrastructure for agricultural AI. Free access to Resourcesat, Cartosat, and RISAT imagery democratizes remote sensing (ISRO, 2023). Value-added products,such as crop classification maps and vegetation indices, reduce technical expertise requirements. The forthcoming NISAR mission (NASA-ISRO Synthetic Aperture Radar), planned for 2024, promises high-resolution SAR imagery for soil moisture mapping and crop structure assessment (ISRO, 2023). NISAR delivers 3-10 m SAR resolution, enabling field-scale monitoring to boost yield forecasts and irrigation precision. The Department of Science and Technology (DST) supports agricultural AI through the National Supercomputing Mission providing HPC access, research grants allocating approximately 22 million USD annually, and international cooperation fostering partnerships for knowledge exchange (DST, 2024). These investments ensure India remains at agricultural AI research forefront.

Public-private collaborations demonstrate sustainable deployment models. Microsoft AI for Earth partnerships with ICRISAT and Andhra Pradesh government reached over 1 million farmers, demonstrating 10–30% yield improvements (Microsoft, 2022). IBM Watson collaborated with ITC e-Choupal developing crop advisory systems (IBM, 2021; IBM Research, 2021). Jio Agri platforms integrate AI analytics with connectivity and digital literacy initiatives (Reliance Industries, 2023). Amazon Web Services (AWS) provides cloud infrastructure for AgriStack through government partnerships (AWS, 2023). These co-development models combine government data assets with private-sector capabilities, while knowledge transfer clauses ensure public-sector capacity building rather than commercial vendor dependence (Carolan, 2020).

### 9.3 Funding, implementation challenges, and interoperability

India’s agricultural AI sector receives substantial support, with public funding exceeding USD 1.8 billion across various schemes in 2024–25, alongside nearly USD 420 million in private venture capital invested in Agri-tech during 2023 (NASSCOM, 2024). However, fragmented funding across ministries causes coordination difficulties, duplication risks, and delayed joint planning. Delayed fund releases disrupt start-up cash flows and project timelines. Funding decisions favor low-risk, proven technologies, potentially limiting support for newer innovations and causing India to lag behind global agricultural AI leaders (World Bank, 2021; CABI, 2024).

Implementation challenges persist despite well-designed initiatives. Nearly 60% of villages lack strong internet connectivity despite substantial BharatNet investments (Telecom Regulatory Authority of India, 2023). Digital literacy gaps compound difficulties, with only 38% of farmers possessing necessary smartphone skills for AI-based services (NSSO, 2023). Extension systems struggle as officers serve approximately 1,000 farmers, far exceeding the recommended 1:300 ratio, reducing support for technology adoption (MoA and FW, 2022). Language barriers limit accessibility since most AI tools support only English and Hindi, while millions speak Tamil, Telugu, Bengali, and Marathi (UNESCO, 2022). Interoperability problems plague India’s digital agriculture system. Separate departmental databases hinder comprehensive farmer profiles (NITI Aayog, 2021). Non-standard data formats complicate multi-source information integration. Legacy systems with outdated software slow modern cloud platform integration and delay digital transformation (FAO, 2019). Absent standardized APIs force costly custom connections for every system pair.

Solutions include developing unified API gateways providing standard interfaces, adopting open data standards following ISO and OGC specifications, strengthening state IT teams, creating public-private task forces, and using phased pilot rollouts for coordinated learning and improved agricultural AI systems (FAO, 2019; World Bank, 2021).

### 9.4 CGIAR’S digital innovation and AgriLLM initiatives

CGIAR leads generative AI efforts for global agriculture, especially for smallholder farmers in developing countries. The 2017 Big Data in Agriculture Platform sets up data sharing across Africa, Asia, and Latin America (CGIAR, 2022). Teaming with AI71, they are building AgriLLM, trained on real farmer questions, to launch a practical chatbot by COP30 with region specific advice (CGIAR, 2025). CGIAR’s Digital Transformation Accelerator (2025–2030) leverages AI, machine learning, big data, and digital tools to optimize food, land, and water management (CGIAR-DTA, 2025). It creates a FAIR data ecosystem, making information findable, accessible, interoperable, and reusable, while offering useful digital services to researchers and policymakers. Focused on ethical, inclusive innovation, it attracts major funding to expand solutions and improve agriculture’s efficiency, sustainability, and fairness.

In India, IRRI’s digital innovation drives faster agricultural transformation by making advanced knowledge easily accessible to rice farmers. Tools like the Rice Knowledge Bank, Rice Crop Managers, and SeedCast support data-driven decisions, while IRRI’s rule-based chatbot offers simple, two-way guidance on seed choice, fertilizer use, and crop management. Together, they improve advisory delivery, strengthen extension systems, and support smarter, timely decisions for rice farmers. The chatbot (PaddyMitra) is a digital advisory tool developed by IRRI to provide rice farmers with quick, accurate, and location-specific information. It works through the WhatsApp platform, and guides seed suitability, seed availability, and fertiliser management in both text and audio (Odia) formats. Its two-way, interactive design helps farmers receive timely, personalised support and makes daily crop-management decisions easier. The PaddyMitra chatbot, launched by IRRI, is an AI-driven WhatsApp tool designed to provide Odisha’s (India) farmers with quick, personalised crop advice (Bharti et al., 2025). It includes guidance on seed choice, fertiliser use, pest and disease management, and weather-based recommendations. The chatbot enables farmers to make informed decisions with timely, easy-to-understand information (IRRI News, 2025a).

The International Rice Research Institute (IRRI) recently launched the Global AIHybrid Rice Platform (GAI-HRP). This AI-powered global digital tool rapidly identifies optimal parental combinations for hybrid rice breeding, accelerating the selection. This AI-powered global digital tool rapidly identifies optimal parental combinations for hybrid rice breeding, accelerating the selection of high-yielding F1 hybrids (IRRI News, 2025b). The FAO’s Digital Agriculture Strategy, launched in 2019, promotes the responsible use of AI to advance Zero Hunger while preventing inequality among farmers (Sabio and Lehoux, 2025). It emphasises inclusive innovation, ensuring that smallholders, women, and marginalised communities benefit from technology (FAO, 2022). The FAO Hand-in-Hand Initiative applies geospatial AI for policy planning, integrating satellite, climate, soil, and socioeconomic data to guide targeted agricultural investments across 55 countries (Mehrabi et al., 2021).

## 10 Transformative potential and challenges

Integrating generative AI into agriculture presents new opportunities to address longstanding issues of scale, access, and precision in farm advisory services. However, making this transformation work demands overcoming significant hurdles in technology, institutional support, and social fairness. This section examines how GenAI can change agricultural practices and identifies the challenges that must be addressed for its fair and sustainable use.

### 10.1 Toward equitable and climate resilient agriculture through GenAI

Climate-resilient agriculture has predominantly benefited large commercial farms in developed countries, widening global farming inequalities. In India, where nearly 86% of farmers cultivate less than two hectares (Tata-Cornell Institute, 2025), high costs of sensors, drones, and specialized expertise have excluded smallholders from technological advances. Generative AI bridges this divide by reducing financial and knowledge barriers (Gesser-Edelsburg et al., 2024). Smartphones serve as accessible gateways to digital advisory platforms, eliminating costly hardware investments (Kamal and Bablu, 2023; Kumar et al., 2025). Through widespread mobile connectivity, conversational AI tools deliver timely, localized advice via voice or text in regional languages, transforming climate adaptation strategies into affordable, knowledge-based services.

GenAI systems scale efficiently, delivering farm-specific recommendations using satellite data and localized models (Ojanji and Dhulipala, 2025). There are various functional roles of GenAI in climate smart agriculture (Figure 6). Ensuring equitable access requires supportive policies and community-based approaches involving Krishi Vigyan Kendras, Farmer-Producer Organizations, and women farmers (Chakraborty et al., 2025). Agriculture faces intensifying climate pressures: erratic monsoons, extreme heat, shifting pest geographies, and coastal salinization (Sivakumar, 2020; Prakash, 2024). Generative AI enhances resilience through climate-informed decision support integrating seasonal forecasts with crop models (Li et al., 2024). LLMs translate complex probabilistic forecasts into actionable recommendations explaining El Niño-Southern Oscillation or Indian Ocean Dipole patterns for local crop performance (Sharma et al., 2024). Early warning systems leverage climate suitability modelling for pest outbreaks and disease epidemics (Kim et al., 2025). Adaptive management enables in-season adjustments including modified irrigation timing, pest-control schedules, and dynamic fertilizer application (Park and Choi, 2024). Parametric insurance products indexed to AI predictions facilitate automated claim processing, enhancing financial resilience (Cao et al., 2024).

### 10.2 Quantum computation as a catalyst for gen AI–driven farming

Beyond Generative AI, integrating quantum computation can further improve the efficiency and accuracy of Gen AI–derived agricultural solutions. Recent studies illustrate this convergence: AgriQDL couples variational quantum circuits and Quantum GANs with deep learning to accelerate high-dimensional satellite, soil, and IoT data processing while generating synthetic samples for data-scarce regions (Mary et al., 2025). Similarly, a quantum-enhanced RAG assistant angle-encodes embeddings onto qubits and uses fidelity-based similarity to deliver accurate farming advisories (Bhavana et al., 2025). Compared with purely classical Gen AI, these quantum-assisted models offer faster computation, stronger generalization, and improved scalability for climate-resilient smart farming.

### 10.3 Digital infrastructure requirements for AI in Indian agriculture

Realizing AI’s agricultural potential requires substantial digital infrastructure investments, though significant gaps constrain deployment. India’s network shows progress: approximately 95.15% of 644,131 villages achieved 3G/4G connectivity by April 2024, though 31,000 lack coverage (Ministry of Communications, 2024). BharatNet connected over 2.14 lakh gram panchayats through 6,92,299 km optical fiber (IBEF, 2025). Data costs dropped from 3 USD per GB (March 2014) to 0.1 USD (March 2024) (TelecomTalk, 2024).

**Figure 7:**
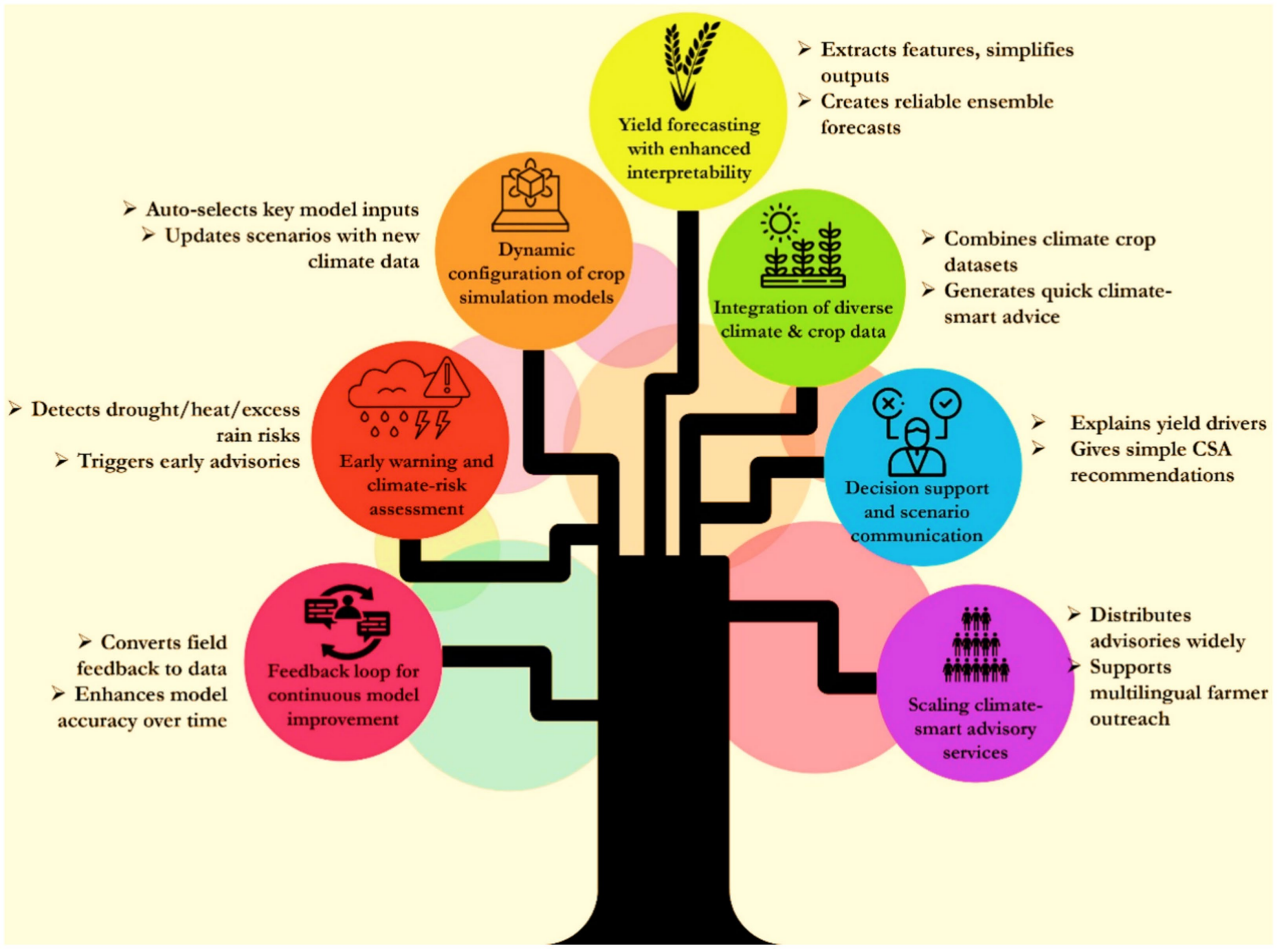
Functional roles of GenAI in climate smart agriculture.

AgriStack offers farmer centric digital infrastructure with three registries: Farmer Registry, Geo Referenced Village Map Registry, and Crop Sown Registry (ICRISAT, 2024). In recent times, Govt. of India has proposed to extend its AI facility to solve and give advisories under a flagship project called Vistaar (Virtually Integrated System to Access Agricultural Resources) (PIB, 2026). Operating across 17 states, 492 districts, and 421,000 villages, AgriStack mapped 253 million plots and generated 60 million Farmer IDs by May 2025 (ICRISAT, 2024). The Ministry announced 665 million USD for registry development (AgroTech Space, 2025). The National Supercomputing Mission deployed 34 supercomputers with 35 Petaflops capacity, supporting over 10,000 researchers (OpenGov Asia, 2025; IBEF, 2022), enabling agricultural simulations (DST, 2024), though underutilized. Human infrastructure is critical. Enhancing AI capacity among 200,000 extension officers supporting 700,000 villages is essential (IndiaAI, 2025a). Research shows 79.25% extension professionals use AI at low levels due to inadequate training (Patel et al., 2024). Maharashtra’s AI Agriculture Policy allocates 5.56 million USD for training (Elets eGov, 2025). Total investment required: 7.2 billion USD. Government earmarked 1,223.8 million USD for IndiaAI Mission over five years (IndiaAI, 2025b). Current trajectory, some of the potential operational barriers and future horizons are given in Figure 7.

### 10.4 Institutional barriers: data silos, model trust, last mile delivery

Institutional challenges often exceed technical limitations. Data silos, trust deficits, and delivery constraints require systemic interventions. Data fragmentation across departments, including land records (Revenue), crop data (Agriculture), weather (India Meteorological Department), and market information (Directorate of Marketing and Inspection), hampers AI effectiveness (MoA and FW, 2023), causing incomplete farmer profiles and lower accuracy. Solutions include mandated data sharing, federated learning, and data trusts. Model trust barriers persist as farmers maintain justified skepticism toward black box AI recommendations, given historical advisory failures and opaque decision making (Posadas et al., 2023). Building trust requires explainable recommendations, farmer testimonials, human review by extension officers, gradual adoption, and clear accountability rules (Dwarampudi and Yogi, 2024). Last mile delivery faces challenges: understaffed extension systems (one officer handling 1,162 versus recommended 750 farmers) (Verma and Bhattacharya, 2018), low digital literacy (14.9% rural internet access, 13% agricultural worker digital literacy) (NITI Aayog, 2019), poor connectivity, and inadequate local language content. Solutions include self-help group support, voice-based tools, offline apps, incentives for agri-input dealers, and video guides via YouTube and WhatsApp (Adithyan et al., 2025). Institutional coordination complexity arises from parallel initiatives across Ministry of Agriculture, Ministry of Electronics, Department of Science and Technology, ISRO, and ICAR (NITI Aayog, 2024). Enhanced coordination through interministerial committees, nodal agencies like NITI Aayog, common performance indicators, and multi-stakeholder consultations can ensure coherence and optimize resources.

Open-source models, federated learning, and community ownership provide pathways for equitable agricultural AI through local customization, decentralized privacy-preserving training, and farmer data ownership via cooperatives. Realizing this requires addressing data quality gaps, bandwidth constraints, and governance challenges while integrating national models with federated systems and cooperative benefit-sharing for sustainable, equitable outcomes.

## 11 Conclusion and roadmap

This current work demonstrates how generative AI is transforming agriculture by evolving beyond simple expert systems to powerful, crop-specific language models that make farming knowledge readily accessible to everyone. Tools like SeedLLM-Rice utilise focused training on agricultural data, working with images from satellites, drones, and smartphones to provide farmers with comprehensive advice that combines visual and textual information. Utilising knowledge graphs and crop simulation models enables AI to gain a deeper understanding of farming and provide more reliable recommendations. Smartphone apps and chatbots enable farmers with limited technical knowledge to receive precise farming advice. India’s National AI Mission and Project Vistaar using AgriStack platform play a significant role in supporting this AI growth at the national and state levels.

**Figure 8:**
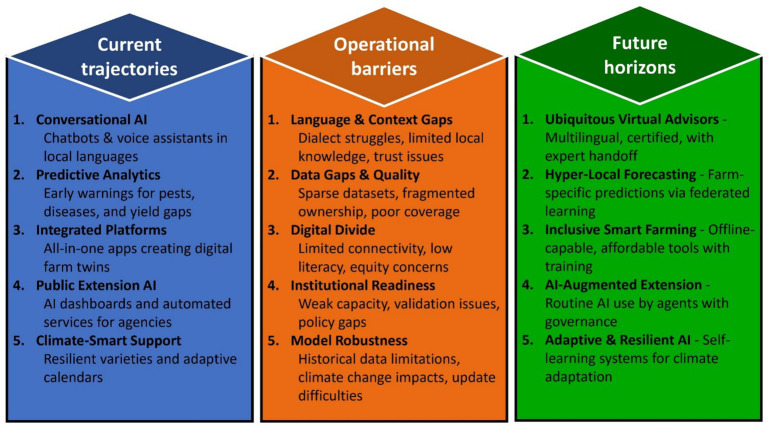
An overview of the present trajectories, operational difficulties, and future scope.

AI in agriculture faces technical limitations, struggling with diverse regions, rare crops, and understanding causal relationships while focusing on single seasons rather than long-term impacts (Figure 8). Critical data gaps persist in rain-fed regions, with traditional knowledge unmapped and farmer records missing over time. Social challenges include insufficient farmer involvement in tool design, unclear benefit distribution, environmental risks like monoculture promotion, weak liability policies, and corporate control limiting farmer autonomy and system openness. The work recommends adopting strict scientific practices, including open data sharing, bias testing, and involving farmers in the research process. Policies should focus on building a strong digital infrastructure, protecting farmers’ data rights, certifying AI tools, training extension workers, and supporting small and marginalised farmers. Developers must create farmer-friendly apps that support multiple languages, work offline, when necessary, respect user privacy, and clearly explain their decision-making processes. Ethical principles and environmental care must guide AI development.

Generative AI, combined with multiple data sources and crop models, can soon offer personalised, fast, and easy-to-use climate-smart advice. However, this comes with risks, such as excluding farmers without internet access, losing traditional knowledge, or allowing a few companies to dominate the market. Significant investments of over $ 6.72 billion USD and coordinated institutions are needed, especially in India, which has a large farming population and ambitious digital plans. Worldwide, agricultural AI must avoid repeating colonial mistakes of exporting solutions without local adaptation. Collaboration among countries in the Global South, cultural adaptation of tools, capacity building, and sharing knowledge openly are vital. Future research should focus on AI that understands causes, models multiple climate stresses, supports biodiversity, accommodates multilingual use, and involves farmers in the design process.
